# The Influence of Growth Rate on ^2^H/^1^H Fractionation in Continuous Cultures of the Coccolithophorid *Emiliania huxleyi* and the Diatom *Thalassiosira pseudonana*

**DOI:** 10.1371/journal.pone.0141643

**Published:** 2015-11-17

**Authors:** Julian P. Sachs, Orest E. Kawka

**Affiliations:** School of Oceanography, University of Washington, Seattle, Washington, 98195, United States of America; Case Western Reserve University, UNITED STATES

## Abstract

The hydrogen isotope (^2^H/^1^H) ratio of lipids from phytoplankton is a powerful new tool for reconstructing hydroclimate variations in the geologic past from marine and lacustrine sediments. Water ^2^H/^1^H changes are reflected in lipid ^2^H/^1^H changes with R^2^ > 0.99, and salinity variations have been shown to cause about a 1‰ change in lipid δ^2^H values per unit (ppt) change in salinity. Less understood are the effects of growth rate, nutrient limitation and light on ^2^H/^1^H fractionation in phytoplankton. Here we present the first published study of growth rate effects on ^2^H/^1^H fractionation in the lipids of coccolithophorids grown in continuous cultures. *Emiliania huxleyi* was cultivated in steady state at four growth rates and the δ^2^H value of individual alkenones (C_37:2_, C_37:3_, C_38:2_, C_38:3_), fatty acids (C_14:0_, C_16:0_, C_18:0_), and 24-methyl cholest-5,22-dien-3β-ol (brassicasterol) were measured. ^2^H/^1^H fractionation increased in all lipids as growth rate increased by 24‰ to 79‰ (div d^-1^)^-1^. We attribute this response to a proportional increase in the fraction of NADPH from Photosystem I (PS1) of photosynthesis relative to NADPH from the cytosolic oxidative pentose phosphate (OPP) pathway in the synthesis of lipids as growth rate increases. A 3-endmember model is presented in which lipid hydrogen comes from NADPH produced in PS1, NADPH produced by OPP, and intracellular water. With published values or best estimates of the fractionation factors for these sources (α_PS1_ = 0.4, α_OPP_ = 0.75, and α_H2O_ = 0) and half of the hydrogen in a lipid derived from water the model indicates α_lipid_ = 0.79. This value is within the range measured for alkenones (α_alkenone_ = 0.77 to 0.81) and fatty acids (α_FA_ = 0.75 to 0.82) in the chemostat cultures, but is greater than the range for brassicasterol (α_brassicasterol_ = 0.68 to 0.72). The latter is attributed to a greater proportion of hydrogen from NADPH relative to water in isoprenoid lipids. The model successfully explains the increase in ^2^H/^1^H fractionation in the sterol 24-methyl-cholesta-5,24(28)-dien-3β-ol from marine centric diatom *T*. *pseudonana* chemostat cultures as growth rate increases. Insensitivity of α_FA_ in those same cultures may be attributable to a larger fraction of hydrogen in fatty acids sourced from intracellular water at the expense of NADPH as growth rate increases. The high sensitivity of α to growth rate in *E*. *huxleyi* lipids and a *T*. *pseudonana* sterol implies that any change in growth rate larger than ~0.15 div d^-1^ can cause a change in δ^2^H_lipid_ that is larger than the analytical error of the measurement (~5‰), and needs to be considered when interpreting δ^2^H_lipid_ variations in sediments.

## Introduction

Discovered in 1931 by Harold Urey [1], deuterium (^2^H) accounts for 0.0156% of hydrogen atoms on Earth, or about one of every 6,420. Since deuterium has twice the mass of protium (^1^H or H), chemical bonds to ^2^H have significantly lower vibrational frequencies than those to H, and as a result, require more energy to break. Reactions involving C-^2^H bonds therefore occur some 5–10 times more slowly than those involving C-H bonds [2,3]. This gives rise to a large kinetic isotope effect and ensuing isotopic fractionations that are much larger than for any other stable isotope system. This characteristic makes the stable hydrogen isotopes particularly sensitive tracers of biological and environmental processes.

Analytical advances in the separation of small molecules by capillary gas chromatography, their pyrolytic reduction to H_2_ gas, and the introduction of that H_2_ into a dual inlet mass spectrometer by a stream of helium by Alex Sessions and others in the 1990s provided a means of precisely (ca. +/- 5‰) measuring ^2^H/^1^H ratios on sub-microgram quantities of individual lipids, or biomarkers [4–6]. Sauer et al. (2001) subsequently demonstrated that the ^2^H/^1^H ratio of microalgal lipids co-varied with that of the water in which the algae grew [7], a relationship borne out by culture studies [8–10].

Because the hydrogen isotopic composition of lake or ocean surface waters is sensitive to local evaporation and precipitation rates [11–13], reconstructions of water isotope variations in the geologic past are possible by measuring ^2^H/^1^H ratios of microalgal lipids in lake or ocean sediment cores [8,14–20]. This technique is analogous to the widely used oxygen isotope method in calcium carbonate microfossils. It can be used where such fossils are non-existent, such as in many lacustrine settings and in parts of the ocean where calcium carbonate is not well preserved. Unlike the oxygen isotopic ratio of biogenic CaCO_3_, which is generally within a few parts per thousand of the water in which the shell was grown after accounting for the effect of temperature, δ^2^H values of lipids are 100‰ to 400‰ depleted in deuterium compared to the water in which the microalgae grew [4,9,21]. What’s more, the ^2^H-depletion in algal lipids varies as a function of taxa [9], lipid type [4,9], growth phase [22,23], and environmental conditions [10,24–27]. This large ^2^H-depletion in algal lipids was presciently attributed by Estep and Hoering (1981) to a very low δ^2^H value of hydride in nicotinamide adenine dinucleotide phosphate (NADPH) [28].

Much of what is known about hydrogen and deuterium cycling within cells comes from ^2^H NMR studies that provide ^2^H/^1^H ratios of individual hydrogen atoms within molecules [29,30], on the one hand, and comparative studies of the hydrogen isotopic composition of different biochemical constituents of plants, microalgae and bacteria under different growth conditions on the other (i.e., autotrophic, heterotrophic and mixotrophic) [28,31,32]. The conclusion common to all these studies is that photosynthetically-produced NADPH is the probable source of the large ^2^H-depletion in lipids relative to environmental water [28,29,31–33].

The other key inference from the ^2^H NMR studies [29,30] and the *Lemna gibba* (duckweed) growth experiments by Yakir and DeNiro (1990) [32] is that rapid and extensive non-enzyme-catalyzed exchange of hydrogen occurs between intracellular water and organic hydrogen. This effect was confirmed and quantified by Kreuzer-Martin et al. (2006 & 2012) [34,35], who measured the hydrogen isotopic composition of intracellular water and fatty acids in bacterial and mammalian cells following growth on ^2^H-labeled water. They concluded that ~50% of the hydrogen in intracellular water had cycled through the organic hydrogen pool in exponentially growing cells, and about half that amount in stationary-phase cells [34,35].

A so-called “vital effect” several times the magnitude of the environmental signal might at first seem untenable for a paleoenvironmental proxy. Yet a growing number of studies continue to find systematic and reproducible variations of algal lipid δ^2^H values not only with water δ^2^H values, but other environmental parameters as well, such as salinity [10,22,24–27,36].

Because a detailed mechanistic understanding of the source of ^2^H-depletion in algal lipids is lacking it is difficult to know *a priori* how particular environmental or growth conditions will manifest in the δ^2^H value of lipids. Through empirical studies performed first by Schouten et al. (2006) [10], it was shown that ^2^H/^1^H fractionation increased as growth rate increased in batch cultures of the marine coccolithophorids *Emiliania huxleyi* and *Gephyrocapsa oceanica*. Growth rates varied in those experiments in response to changes in salinity and temperature. The extent to which salinity and/or temperature themselves caused the observed changes in ^2^H/^1^H fractionation, independent of changes in growth rate, was difficult to diagnose. Furthermore, in a batch culture, the biomass, light intensity, nutrient concentrations, waste products, and consequently the cellular environment, are always changing. Motivated by the high sensitivity of ^2^H/^1^H fractionation to growth rate implied by the Schouten et al. (2006) experiments [10], we set out to quantify the ^2^H/^1^H fractionation response in coccolithophorid and diatom lipids to changes in growth rate using continuous culturing techniques that permit controlled, steady-state growth to be maintained. The first of these results were published by Zhang et al. (2009) [37] based on just two continuous cultures of the diatom *Thalassiosira pseudonana*. They observed greater ^2^H/^1^H fractionation at the higher growth rate in the sterol 24-methyl-cholesta-5,24(28)-dien-3β-ol but not in three fatty acids. The results from *T*. *pseudonana* in the present study generally confirm the earlier findings, but slight differences in culturing conditions preclude a direct comparison.

We find that ^2^H/^1^H fractionation in alkenones, fatty acids and a sterol from the coccolithophorid *Emiliania huxleyi*, and a sterol from the marine diatom *Thalassiosira pseudonana* increases as growth rate increases, by 20–79‰ (div d^-1^)^-1^. We attribute this large and systematic hydrogen isotope effect on lipids from growth rate to (i) changing contributions of NADPH from photosynthesis on the one hand, and the oxidative pentose phosphate pathway and tricarboxylic acid cycle on the other, as well as (ii) the relative contributions of hydrogen from intracellular water and NADPH, and potentially, (iii) changes in the ^2^H/^1^H ratio of intracellular water as growth rate changes.

## Materials and Methods

### Culture Methods

#### *E. huxleyi*

Cultures of *E*. *huxleyi* (CCMP Strain 374) were obtained from the Provasoli-Guillard National Center for Marine Algae and Microbiota (NCMA), formerly the National Center for Culture of Marine Phytoplankton (CCMP). Information provided by NCMA indicates strain collection on 6/23/1989 from a water depth of 5 m in the Gulf of Maine area by P. Holligan with isolation and deposition at CCMP by T. Skinner (9/1/1990 and 10/22/90, respectively). The axenic culture is maintained at NCMA at 18–22°C in f/2-Si or L1-Si media.

Growth media for the batch and four continuous cultures of *E*. *huxleyi* utilized seawater collected from Puget Sound between October, 2006 and June, 2007 from a range of locations bounded by the Straits of Juan de Fuca on the north and Elliot Bay on the South. Seawater salinity in these collections ranged between 30.19 and 31.37, with an average of 30.70. Nutrient composition of the stock seawater was as follows (average ± 1 SD, for N = 4): 2.40 ± 0.21 μM PO_4_^3-^, 27.49 ± 3.36 μM NO_3_^1-^, 0.25 ± 0.21 uM NO_2_^1-^, 2.24 ± 1.91 μM NH_4_^1+^, and 70.06 ± 27.00 μM Si(OH)_4_ (aq). The seawater was sterilized by filtration through Millipore 0.45 um Type HA filters followed by autoclaving. Growth media for the two continuous cultures under nutrient replete (NR) conditions used this seawater enriched as per the f/2-Si (silicate addition excluded) formulation [38,39] resulting in a molar N/P ~ 24:1 in the feed media. In order to establish N-limited (N2L) growth rates in two continuous cultures, nitrate and phosphate additions were modified from the f/2-Si recipe to attain a molar N/P ratio of <1.5. The calculated concentrations of nitrogen in the growth (feed) media and the associated N:P ratios for all four *E*. *huxleyi* continuous cultures are summarized in Table 1.

**Table 1:** Growth conditions for continuous cultures of *E*. *huxleyi* and *T*. *pseudonana*.

Media Type	Number of Generations	Division Rate (div day^-1^)	Cell Density (cells mL^-1^) /10^6^	Feed Media Nitrate (μM)	Feed Media Molar N/P Ratio	Residual Nitrate (μM)	Residual Phosphate (μM)	Residual Molar N/P Ratio
*E*. *huxleyi*								
N2L	3.53	0.20	n.d.	30.5	1.42	3.03 ± 0.07 (2)	11.85 ± 0.01 (2)	0.256 ± 0.006 (2)
N2L	4.01	0.69	n.d.	24.6	1.16	n.d.	n.d.	n.d.
NR	4.44	0.89	n.d.	958	23.7	n.d.	n.d.	n.d.
NR	4.09	0.99	n.d.	951	23.6	n.d.	n.d.	n.d.
*T*. *pseudonana*								
N2L	4.20	0.52	2.08 ± 0.20 (8)	98.0	5.18	0.04 ± 0.05 (7)	1.34 ± 0.25 (7) ^†^	0.034 ±0.046 (7) ^†^
N2L	5.08	1.41	1.95 ± 0.10 (5)	98.0	5.18	0.60 ± 0.25 (4)	1.76 ± 0.32 (4) ^†^	0.346 ± 0.153 (4) ^†^
N2L	5.81	2.07	1.73 ± 0.13 (4)	98.0*	5.18*	6.66 ± 1.98 (3)	7.60 ± 0.56 (3)	0.870 ± 0.211 (3)

Cell densities for *T*. *pseudonana* are presented as average ± SD (number of samples), where each sample represents the density measured on the days before harvesting. The symbol n.d. means no data available. The feed media nitrate concentrations and N/P ratios were calculated from the mass of nutrient salts added to the media.

* The measured nitrate concentration and molar N/P ratio for the N2L feed media in this *T*. *pseudonana* culture were 96.79 ± 2.77 μM and 5.81 ± 0.07, respectively, close to the values determined from based on the mass of nutrient salts added to the media.

^†^ These residual phosphate concentrations and associated molar N/P ratios represent minimum and maximum values, respectively, owing to the potential loss of phosphate during sample storage (as discussed in Materials and Methods: Culture Methods: *T*. *pseudonana*).

Upon receipt from NCMA, the *E*. *huxleyi* strain was revived by batch culturing with f/2-Si media (NR) in capped 25 mL glass culture tubes under light and temperature conditions representative of those to be used for continuous cultures. In vivo fluorescence measurements were used to monitor biomass increase in the batch cultures. Sequential and multiple transfers of the strain to fresh media by subsampling and inoculation during exponential phase of growth ensured acclimation and a valid determination of the maximum growth rate attainable under the provided conditions. With batch culturing in NR media, this CCMP Strain 374 of *E*. *huxleyi* attained a maximum growth rate of 1.31 ± 0.05 div d^-1^ (specific growth rate of 0.91) for N = 4 samples.

All tubing and culture vessels used in this study were sterilized by autoclaving. The continuous cultures of *E*. *huxleyi* were grown at ~ 20°C in 15L polycarbonate culture vessels illuminated continuously by four 48” Cool White Fluorescent 40 Watt Bulbs and gently stirred (50 to 60 rpm) with either a Lightning stirrer with impeller or a Nalgene Magnetic Carboy Stirrer coupled with a magnetic stir plate. Light intensity in the growth chamber area, based on previous measurements in our similarly illuminated studies, was 200 ± 20 μmol m^-2^ s^-1^ [37]. The cultures were supplied medical grade air by gentle bubbling just below the media surface. Sterile growth media of either type NR or N2L was fed to the culture vessel using a peristaltic pump, and the media volume (7 L) in the culture vessel was kept constant by positioning a media withdrawal tube at an appropriate height in the vessel and connected to a second peristaltic pump.

For *E*. *huxleyi*, each of the four continuous cultures was inoculated with algae from either the NR or N2L batch culture; and the respective population was allowed to reach a healthy cell density, as indicated by significant in vivo fluorescence, before the peristaltic pumps were turned on. Under steady-state conditions of nutrient-limited growth rate and constant algal cell density (ln of fluorescence), the specific growth rate (d^-1^) is equivalent to the dilution rate D of the culture, ie. D = F_m_/V_c_ where F_m_ and V_c_ are the inflow of the media and the volume of the culture, respectively. The cell division rate, the unit of growth rate used in this study, is provided by D/ln(2). The media feeds were set to provide a range of nutrient-limited growth rates by adjusting the inflow peristaltic pump. *E*. *huxleyi* cell division rates achieved in this study were 0.99 and 0.89 div d^-1^ for the NR-based cultures, and 0.69 and 0.20 div d^-1^ for the N2L-based cultures (Table 1).

The inflows to the culture vessel were measured daily and the inflow peristaltic pump was adjusted to keep a constant dilution rate. Simultaneous monitoring of in vivo fluorescence on samples of the continuous culture withdrawn daily provided an estimate of cell density and the degree of stabilization of the algal population at a specific growth rate. Once the cell density stabilized, the continuous culture was allowed to run a minimum of 3.5 generations, after which the algal culture was harvested.

Comparison of the nitrate (3.03 μM) and phosphate (11.85 μM) concentrations measured in the culture media of the lowest growth rate (0.20 div d^-1^) *E*. *huxleyi* continuous culture at steady-state represent 10- and 2-fold reductions from their concentrations in the feed media, respectively, and a reduction in molar N/P ratio from 1.4 to 0.26 (Table 1).

#### *T. pseudonana*

The *T*. *pseudonana* cultures were obtained from the School of Oceanography, University of Washington [37]. Growth media for the batch and three (3) continuous cultures of this second species utilized artificial seawater made by dissolving sea salt (Instant Ocean^**®**^ Aquarium Mixture) in Milli-Q water to attain a salinity of 32. The measured nutrient composition of the artificial seawater was: 0.03 μM PO_4_^3-^; 1.11 μM NO_3_^1-^; 0.15 uM NO_2_^1-^; 0.36 μM NH_4_^1+^; and 4.14 μM Si(OH)_4_. The seawater was gravity-filtered through a 142 mm GF/F filter followed by a 0.8/0.2 μm Supor^**®**^ AcroPak^™^ cartridge filter (Pall Life Sciences). Both NR and N2L versions of the feed media were based on the f/2 medium [38,39] with component stock solutions added sterilely by syringe outfitted with a 25 mm 0.2 um SFCA syringe filter (Thermos Scientific^**™**^ Nalgene^**™**^). The macronutrient enrichments were modified from the f/2 medium recipe to provide the following nominal concentrations for the NR medium: 38 μM PO_4_^3-^, 891 μM NO_3_^1-^, 106 μM Si(OH)_4_; and the N2L medium: 19 μM PO_4_^3-^, 98 μM NO_3_^1-^, 201 μM Si(OH)_4_. This provided molar N/P ratios of 23 and 5.2 for the NR and N2L media, respectively.

Batch culturing of *T*. *pseudonana* in the NR media described above was used to sustain the culture until chemostat inoculation. The highest growth rate observed in NR media was 2.98 div d^-1^ for n = 3 samples, which is comparable to the previously reported rate of 2.89 div d^-1^ with a different growth media [37] (see S1 Appendix).

All three continuous cultures of *T*. *pseudonana* used the N2L formulation of diatom-required media, but the same illumination, similar growth temperatures (20 to 22°C) and similar inoculation, maintenance, and sampling protocols as those described for E. *huxley*i. The flow rate of the N2L feed media was adjusted to obtain *T*. *pseudonana* cell division rates of 2.07, 1.41, and 0.52 div d^-1^ for culture volumes of 6.8, 6.8, and 9.8 L, respectively, and the cells were harvested after 4.2 to 5.8 generations of steady-state conditions (constant fluorescence and cell density). The cell densities observed during steady-state were 1.73, 1.95, and 2.08 x 10^6^ cells mL^-1^, respectively (Table 1).

The residual concentration of nitrate (6.66 μM) and phosphate (7.60 μM) in the *T*. *pseudonana* continuous culture media at the highest division rate (2.07 div d^-1^) represented a 15-fold reduction from their concentrations in the feed media, whereas this reduction was 2.5-fold in the slowest growing (0.52 div d^-1^) chemostat (Table 1). This resulted in a reduction of the molar N/P ratios from 5.2 to 0.87 in the highest growth-rate culture (Table 1).

Residual nutrients in the two lowest growth rate *T*. *pseudonana* cultures were measured after long-term storage of the frozen samples. Clementson and Wayte (1992) reported that while nitrate concentration exhibited no significant change in concentration after 24-months of frozen storage, phosphate steadily decreased after 4 months [40]. In this study, comparison of replicate residual nutrient samples of the 2.07 div d^-1^ culture confirmed the likely loss of phosphate but preservation of the nitrate during long-term freezing. Therefore, while the residual phosphate concentrations of 1.34 and 1.76 μM in the two lowest growth rate cultures should be considered minimum amounts, the residual nitrate concentrations are representative.

### Lipid extraction, purification and derivatization

The phytoplankton cells were isolated from the continuous cultures by either gravity or vacuum-assisted gravity filtration through pre-combusted 142 mm Whatman GFF filters (GE Healthcare Bio-Sciences, Pittsburgh, Pennsylvania, USA). The filters were frozen and kept at ≤ -10°C until further subsampling and analysis.

Lipids were extracted from the freeze-dried filter subsamples utilizing a Accelerated Solvent Extraction System 200 (Dionex ASE-200, Thermo Scientific, Sunnyvale, California, USA) and a 9:1 (v/v) mixture of dichloromethane:methanol as extraction solvent.

The lipids were separated into a non-polar (NP) and a polar, fatty-acid containing fraction (FA) using solid-phase extraction (SPE) on an aminopropyl column. The NP fraction contained the long-chain alkenones, alkenoates, and the 24-methyl cholest-5,22-dien-3β-ol sterol (brassicasterol) from the *E*. *huxleyi* and the 24-methyl-cholesta-5,24(28)-dien-3β-ol sterol from the *T*. *pseudonana* culture extracts. The latter sterol was isolated in sufficiently pure form for hydrogen isotope analysis using further separation of the appropriate NP fraction on an SPE column packed with 5% deactivated silica gel.

Long-chain di- and tri-unsaturated C_37_ and C_38_ alkenones and brassicasterol were isolated from the NP fractions of the *T*. *pseudonana* extracts by semi-preparative high-performance liquid chromatography—mass spectrometry (HPLC-MS) using the alkenone purification method previously described [41] modified to facilitate simultaneous isolation of brassicasterol and for optimal utilization of NP lipid SPE fractions from algal culture extracts (as described in the S2 Appendix).

The fatty acids were methylated for both quantification and hydrogen isotope analysis using methanolic HCl The contribution of introduced methyl group hydrogens to the δ^2^H values of the fatty acids was determined by simultaneous methylation of a Na-phthalate standard of known δ^2^H value (-95.5 ± 2.2, Dr. Arndt Schimmelmann, Indiana University). The fatty acid δ^2^H values reported herein have been corrected for the added methyl hydrogens of δ^2^H = 156.2, and represent those of the free carboxylate anions.

Aliquots of each NP SPE fraction containing the alkenones and brassicasterol and each silica-gel SPE subfraction containing the 24-methyl-cholesta-5,24(28)-dien-3β-ol were silylated (BSTFA + 1% TMCS), after addition of an internal standard (5α-cholestane), to allow for quantification of the alkenones and sterols by GC-FID.

Derivatization of sterols for hydrogen isotope analysis requires a product highly resistant to decomposition and the ability to correct the δ^2^H of the sterol for hydrogens added, neither of which is adequately provided by silylation. The HPLC isolates of brassicasterol and the SPE fraction containing the 24-methyl-cholesta-5,24(28)-dien-3β-ol were acetylated using acetic anhydride with a known δ^2^H value of -133.2 ± 2.1‰ (Dr. Arndt Schimmelmann, Indiana University). The hydrogen isotope values reported for the sterols have been corrected for the hydrogens added by the acetyl group and represent the sterols without the hydroxyl hydrogen atom.

Detailed descriptions of the lipid extraction, purification, and derivatization methods are available in the S2–S4 Appendices.

### GC-FID and GC-MS analyses

The alkenone, sterol, and fatty acid compositions of the continuous cultures were evaluated and quantified by gas chromatography—flame ionization detection (GC-FID) by comparing their area responses with that of the added 5α-cholestane internal standard. Quantification of fatty acids (as methyl esters) proceeded similarly but were additionally corrected for procedural recovery (~70%) using a known mass of heneicosanoic acid added to the culture extract before SPE.

Analysis of the HPLC-isolated alkenones and test fractions by GC-FID, with 5α-cholestane internal standard added, provided confirmation of adequate HPLC purification and appropriate dilution amounts for subsequent hydrogen isotope analyses by GC-IRMS. Silylation of test fractions bracketing the HPLC elution time of brassicasterol confirmed that the combined vials contained all of the compound. Aliquots of the acetylated sterols and methylated fatty acids were similarly quantified to determine the mass isolated and dilution required for subsequent GC-IRMS analysis.

Gas chromatography—mass spectrometry (GC-MS) analysis was used for compound identifications, qualitative analysis of the HPLC fractions, and confirmation of GC-FID results.

Additional details of the GC-FID and GC-MS methods are available in the S5 Appendix.

### Hydrogen isotope analysis of lipids and water

The stable hydrogen isotopic compositions of the purified alkenones, brassicasterol, 24-methyl-cholesta-5,24(28)-dien-3β-ol and fatty acids were determined using gas chromatography—isotope ratio mass spectrometry (GC-irMS; instrument details in the S6 Appendix).

The H_3_^+^ factor was determined daily or between batches of runs using 10 injections of H_2_ reference gas of known δ^2^H, and this correction typically ranged from 4 to 6. Consistent operation of the irMS component of the system was ensured by monitoring its response to the reference gas injected at the beginning and end of each analysis. The overall performance of the combined GC-irMS and consistency in δ^2^H measurements was ensured by daily injections of a series of *n*-C_14_ to *n*-C_44_ *n*-alkanes of known hydrogen isotope composition (Schwab and Sachs, 2009) [41] and instrument maintenance was scheduled accordingly. Hydrogen isotope data was processed using ISODAT 2.5 software.

In order to quantitatively compensate for the potential drifts in measured δ^2^H values as a result of changes in GC elution time, column aging, thermal conversion efficiency, and instrument drift and/or memory effects during and between analyses, standards of known δ^2^H that bracket the retention times of the target compounds were coinjected. For the alkenones, *n*-C_38_ and *n*-C_41_ *n*-alkanes of known δ^2^H (-99.4‰ and -205.7‰, respectively; Dr. Arndt Schimmelmann, Indiana University) were coinjected. The n-C_41_ coinjection standard was used to correct the measured δ^2^H values via reprocessing with the onboard ISODAT software. The *n*-C_38_ coinjection standard was used only for a secondary quality control check (ISODAT calculated δ^2^H = -100.1 ± 5.1‰ for n = 27 sample runs). Coinjection of *n*-C_32_ and n-C_36_ *n*-alkanes of known δ^2^H (-225.9‰ and -212.7‰, respectively; [41] was used for similarly correcting the brassicasterol and the 24-methyl-cholesta-5,24(28)-dien-3β-ol δ^2^H values. Analogous corrections of the fatty acid measurements utilized coinjection of *n*-C_14_ and *n*-C_26_ *n*-alkanes of known δ^2^H (-68.8‰ and -57.7‰, respectively; Dr. Arndt Schimmelmann, Indiana University). Nominally, triplicate injections were averaged to arrive at the final δ^2^H values, with the values reported for brassicasterol and fatty acids corrected for hydrogen added during derivatization.

The δ^2^H values of the media water for the continuous cultures were obtained using a Thermo Finnigan High Temperature Conversion Elemental Interface (TC/EA) equipped with a CTC Analytics GCPal Autosampler and interfaced with the Delta V Plus irMS (described in the S6 Appendix). Thermal conversion of the water was conducted at a pyrolysis temperature of 1450°C. Six replicate analyses of each media sample allowed minimization of any memory effect of the system by exclusion of the first three samples from the final average. Measured values were referenced to Vienna Standard Mean Ocean Water (VSMOW) by calibration with a combination of secondary water standards and VSMOW, Greenland Ice Sheet Precipitation (GISP), and Standard Light Antarctic Precipitation (SLAP) primary standards.

## Results

### *E. huxleyi*

**Fig 1:**
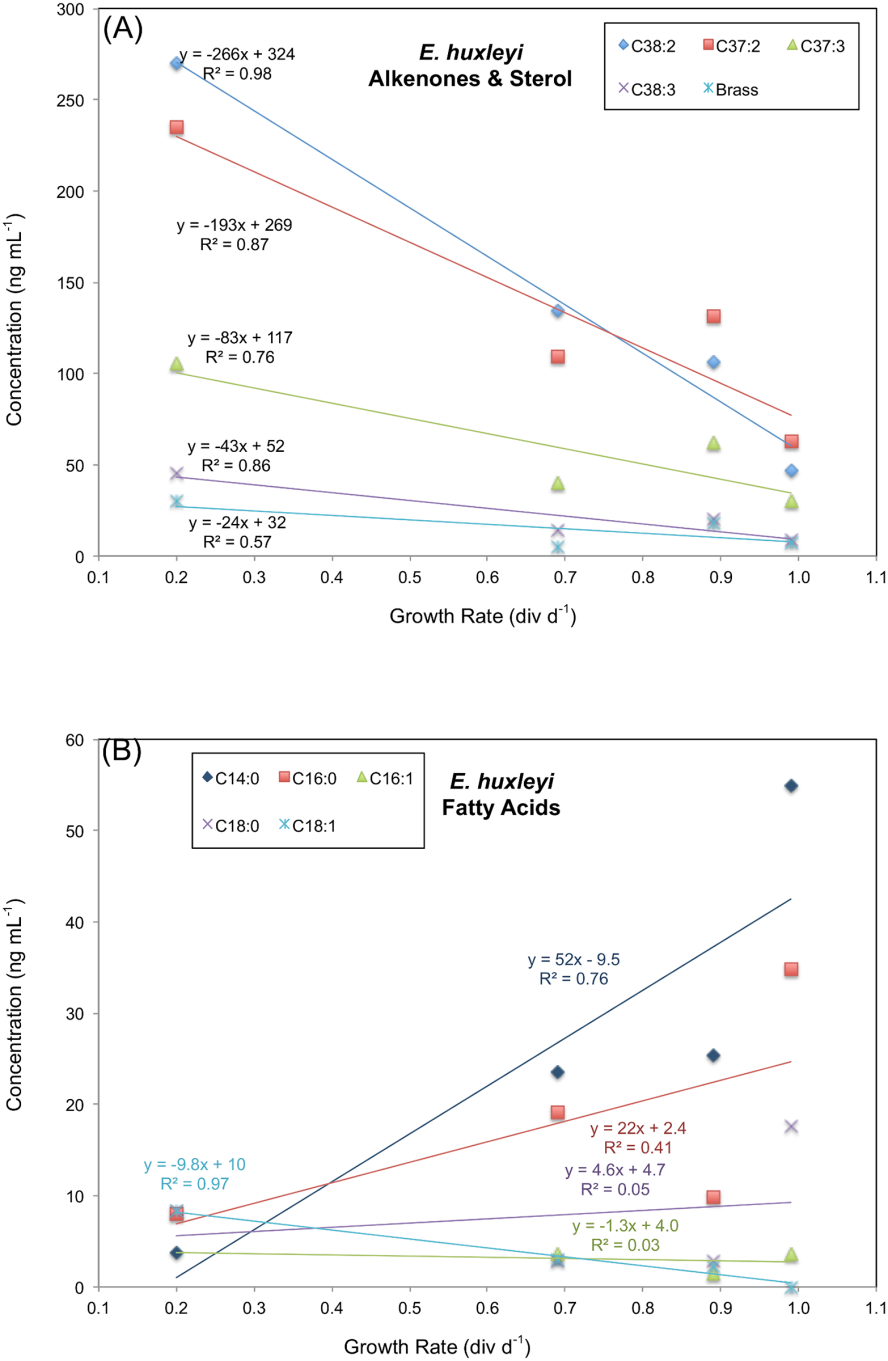
Concentration of lipids as a function of growth rate in *E*. *huxleyi* cultures. Concentrations presented in ng lipid per mL of culture media. (A) Alkenones and brassicasterol. (B) Fatty acids.

**Fig 2:**
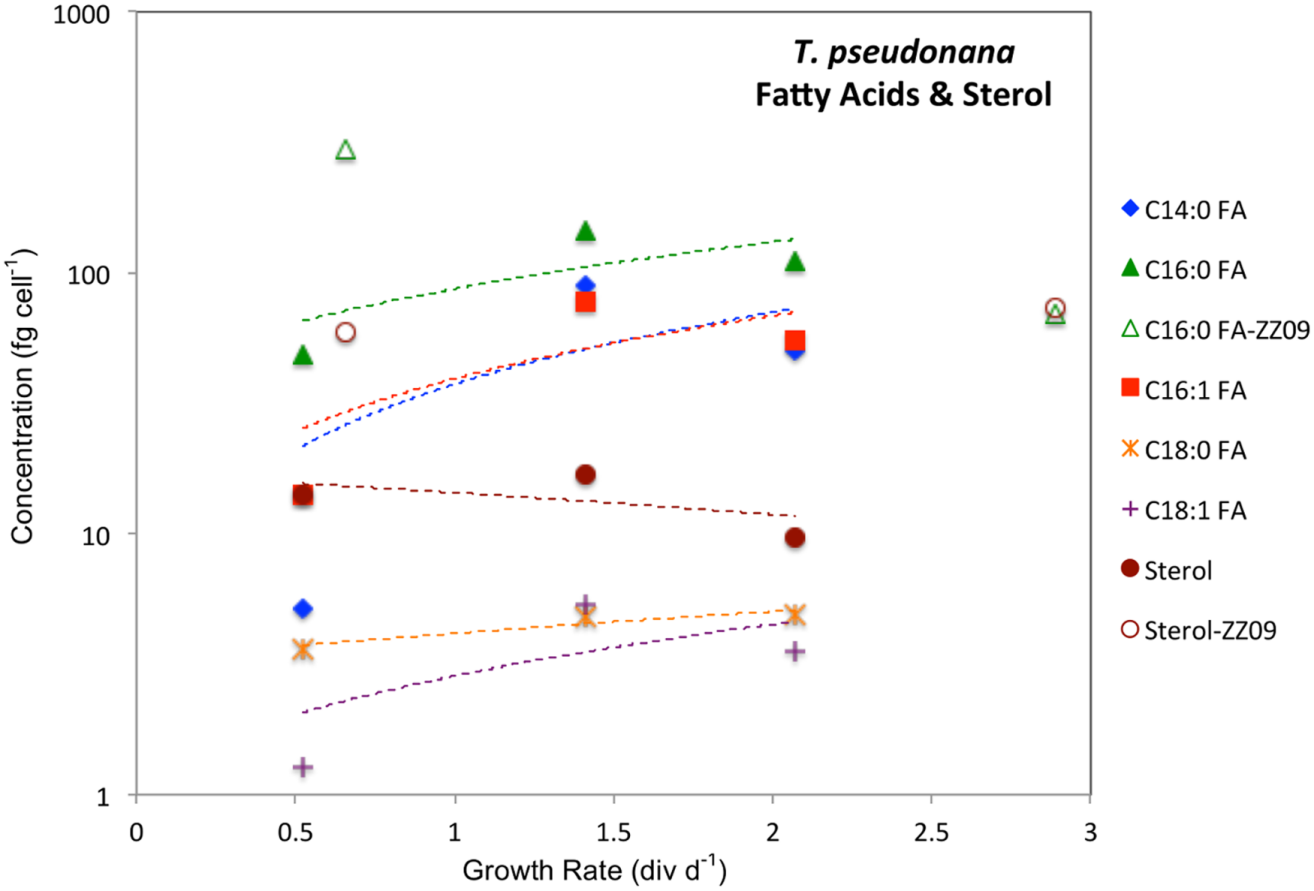
Concentration of lipids as a function of growth rate in *T*. *pseudonana* cultures. Concentrations presented in 10^−15^ g (fg) cell^-1^. Open symbols represent estimates from [37]. Best fit lines in are curved owing to the log scale of the y-axis. The purpose of fitting lines to 3 data points is to demonstrate the positive slope for FAs and negative slope for 24-methyl-cholesta-5,24(28)-dien-3β-ol.

The marine coccolithophorid *Emiliania huxleyi* (CCMP Strain 374) was grown in continuous cultures at 0.99, 0.89, 0.69 and 0.20 div d^-1^ (Table 1). All conditions were held constant between the four treatments except for the rate at which fresh media was supplied and the nitrate-to-phosphate ratio of that media (Table 1). The concentration of the primary sterol (24-methyl cholest-5,22-dien-3β-ol, or brassicasterol), five fatty acids (myristic (C_14:0_), palmitic (C_16:0_), palmitoleic (C_16:1_), stearic (C_18:0_), oleic (C_18:1_)) and four alkenones (methyl ketones: C_37:2_, C_37:3_, C_38:2_, C_38:3_) in the cultures are given in Table 2. Alkenone concentrations varied between 9 and 270 ng mL^-1^ of culture and decreased as growth rates increased (Fig 1A). Brassicasterol concentrations were between 5 and 30 ng mL^-1^ of cultures and decreased as growth rates increased (Fig 1A). Fatty acid concentrations were between 2 and 55 ng mL^-1^ of culture and did not vary systematically with growth rate, with two increasing (C_14:0_, C_16:0_), one decreasing (C_18:1_), and two showing no trend (C_16:1_ and C_18:0_) (Fig 1B). As discussed below lipid concentrations are reported on a per-cell-basis for *T*. *pseudonana* cultures (Fig 2).

**Table 2:** Lipid concentrations in *E*. *huxleyi* chemostat cultures.

		Fatty Acids (ng mL^-1^)					Alkenones (ng mL^-1^)					
Growth Rate (div d^-1^)	Sterol* (ng mL^-1^)	C_14:0_	C_16:1_	C_16:0_	C_18:1_	C_18:0_	C_37:2_	C_37:3_	C_38:2_	C_38:3_	U^k'^_37_	U^k’^_37_-SST (°C)
0.20	30.1	n.d.	n.d.	n.d.	n.d.	n.d.	235	105	270	45.6	0.690	19.2
0.69	5.09	23.5	3.6	19.1	2.9	2.8	109	40.0	135	14.3	0.733	20.4
0.89	17.8	25.3	1.5	9.9	2.3	2.8	131	61.9	106	19.9	0.680	18.8
0.99	7.67	54.9	3.7	34.9	n.d.	17.7	62.8	30.2	46.9	9.01	0.675	18.7

* Brassicasterol.

Concentrations of lipids in *E*. *huxleyi* chemostat cultures in ng per mL culture media. Alkenone unsaturation ratios (U^k’^_37_) and inferred SST based on the Prahl et al. temperature calibration [42] are also provided. The symbol n.d. means no data available. Lipid concentration per cell was not calculated owing to a lack of cell counts.

Hydrogen isotope ratios of lipids in the *E*. *huxleyi* chemostat cultures were between –337‰ for brassicasterol at 0.99 div d^-1^ and –188‰ for C_18:0_ at 0.2 div d^-1^ and generally decreased with increasing growth rate. δ^2^H values for lipids are listed in Table 3, except for C_16:1_ and C_18:1_, the concentrations of which were too low for δ^2^H analyses. Water δ^2^H values in the four cultures were -9.2‰, -6.4‰, -4.5‰, and -6.5‰, respectively, for the growth rates of 0.2, 0.69, 0.89, and 0.99 div d^-1^ (Table 3). δ^2^H values were generally highest for fatty acids (-254‰ to -188‰), intermediate for alkenones (-231‰ to -196‰), and lowest for brassicasterol (-298‰ to -337‰) at any particular growth rate. δ^2^H values were lower for tri-unsaturated alkenones than for di-unsaturated alkenones.

**Table 3:** Hydrogen isotope ratios and fractionation factors in *E*. *huxleyi and T*. *pseudonana* chemostat cultures.

Lipid	Growth Rate(div d^-1^)	δ^2^H -H_2_O	SD-H_2_O	δ^2^H -Lipid	SD-Lipid	α	SD-α	N
*E*. *huxleyi*								
	0.2	-9.2	0.4	-196	7.41	0.811	0.00748	3
C37:2	0.69	-6.4	1.4	-219	1.59	0.786	0.00160	4
	0.89	-4.5	0.4	-214	3.18	0.789	0.00320	4
	0.99	-6.5	0.4	-227	4.65	0.778	0.00468	3
	0.2	-9.2	0.4	-207	2.43	0.800	0.00245	3
C37:3	0.69	-6.4	1.4	-221	5.52	0.784	0.00555	3
	0.89	-4.5	0.4	-216	2.80	0.787	0.00281	3
	0.99	-6.5	0.4	-227	6.88	0.778	0.00693	5
	0.2	-9.2	0.4	-196	2.81	0.811	0.00284	3
C38:2	0.69	-6.4	1.4	-211	3.48	0.794	0.00350	3
	0.89	-4.5	0.4	-210	3.86	0.794	0.00388	3
	0.99	-6.5	0.4	-212	3.52	0.793	0.00355	3
	0.2	-9.2	0.4	-207	2.21	0.800	0.00223	3
C38:3	0.69	-6.4	1.4	-218	4.19	0.787	0.00422	3
	0.89	-4.5	0.4	-225	13.1	0.778	0.0132	3
	0.99	-6.5	0.4	-231	11.7	0.774	0.0118	3
Brassi	0.2	-9.2	0.4	-298	2.90	0.719	0.00275	3
caster	0.69	-6.4	1.4	-312		0.703		1
ol	0.89	-4.5	0.4	-319	2.18	0.695	0.00206	3
	0.99	-6.5	0.4	-337	7.15	0.681	0.00675	3
	0.2	-9.2	0.4	-197	5.76	0.810	0.00712	3
C14:0	0.69	-6.4	1.4	-246	5.06	0.759	0.00588	4
FA	0.89	-4.5	0.4	-252	1.70	0.751	0.00209	3
	0.99	-6.5	0.4	-254	2.34	0.751	0.00258	6
	0.2	-9.2	0.4	-193	20.4	0.814	0.0252	3
C16:0	0.69	-6.4	1.4	-225	7.25	0.781	0.00789	7
FA	0.89	-4.5	0.4	-224	11.4	0.779	0.0126	6
	0.99	-6.5	0.4	-232	7.86	0.773	0.00846	8
	0.2	-9.2	0.4	-188	17.7	0.819	0.0253	2
C18:0	0.69	-6.4	1.4	-191	9.29	0.814	0.0105	5
FA	0.89	-4.5	0.4	-201	4.38	0.802	0.00508	4
	0.99	-6.5	0.4	-214	6.48	0.791	0.00697	8
*T*. *pseudonana*								
	0.52	-75.2	0.36	-366	8.8	0.685	0.0095	3
Sterol	1.41	-74.3	0.46	-387	4.0	0.662	0.0043	3
	2.07	-74.5	0.37	-394	3.6	0.655	0.0038	3
C14:0	0.52	-75.2	0.36	-234	3.18	0.828	0.0034	3
FA	1.41	-74.3	0.46	-252	3.23	0.808	0.0034	3
	2.07	-74.5	0.37	-243	2.71	0.818	0.0028	3
C16:0	0.52	-75.2	0.36	-238	1.86	0.824	0.0018	3
FA	1.41	-74.3	0.46	-237	3.26	0.825	0.0034	3
	2.07	-74.5	0.37	-222	4.65	0.841	0.0049	3
C16:1	0.52	-75.2	0.36	-223	4.34	0.840	0.0046	3
FA	1.41	-74.3	0.46	-224	4.03	0.838	0.0043	3
	2.07	-74.5	0.37	-214	1.36	0.849	0.0013	3

SD = standard deviations of the tabulated averages. N = number of samples averaged. δ^2^H_lipid or H2O_ = [(^2^H/^1^H) _lipid or H2O_ − (^2^H/^1^H) _std_]/(^2^H/^1^H) _std_*1000 with VSMOW as reference standard. α = (δ^2^H _lipid_ + 1000)/(δ^2^H _H2O_ + 1000).

Fractionation factors (α = (δ^2^H_lipid_ + 1000)/ (δ^2^H_H2O_ + 1000)) were determined using the individual compound-specific δ^2^H_lipid_ measurements and an average value for the δ^2^H_H2O_ of the respective culture water (see formulas in Table 3). The averages of these individual α values along with their associated standard deviations (**SD-**α) are presented in Table 3.

**Fig 3:**
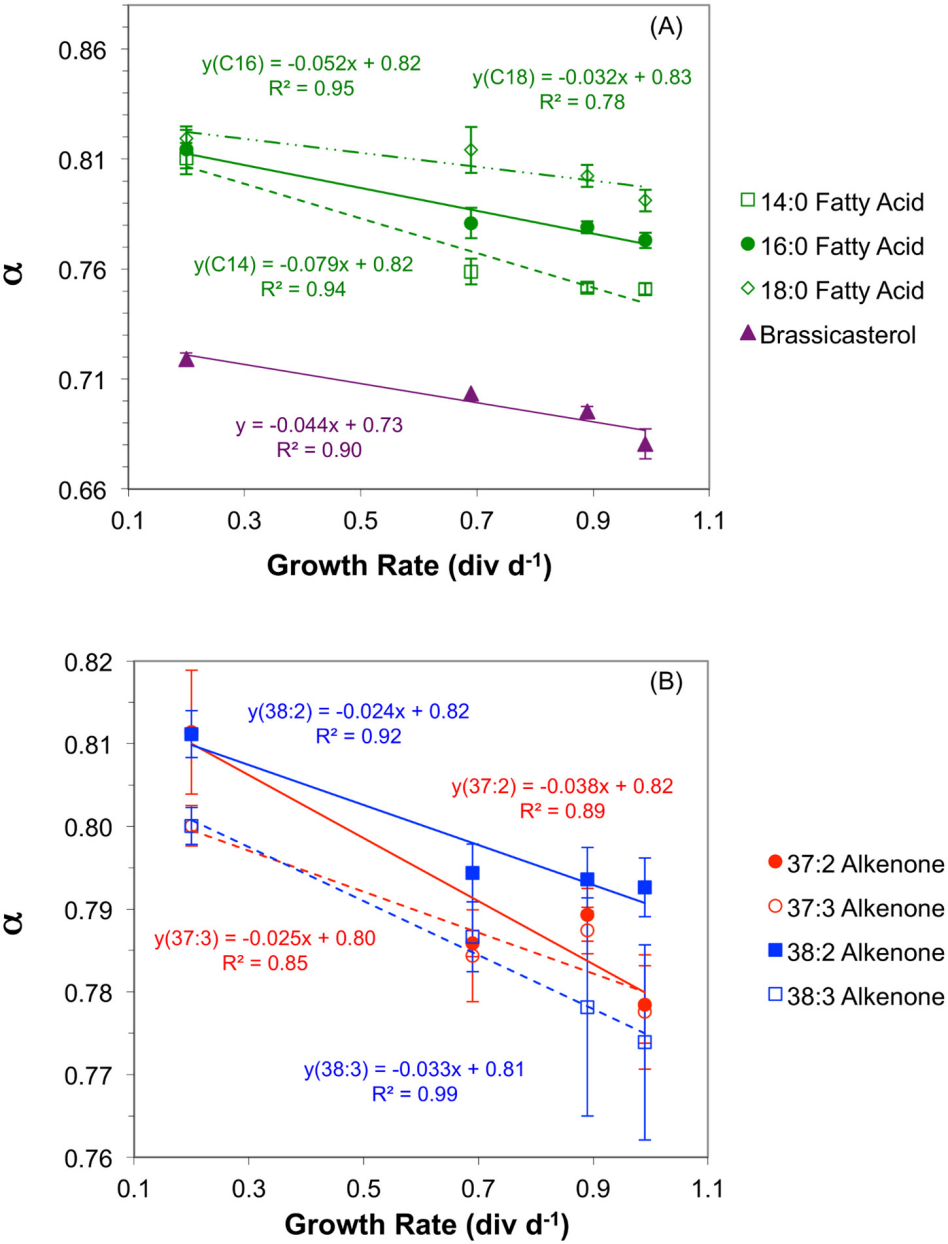
Hydrogen isotope fractionation in lipids as a function of growth rate in *E*. *huxleyi* chemostat cultures. (A) Fractionation factors (α) decreased, indicating greater ^2^H/^1^H fractionation between lipids and extracellular water, as growth rates increased by 44‰ (div d^-1^)^-1^ for brassicasterol, and 79‰ (div d^-1^)^-1^ for myristic acid (C_14:0_), 52‰ (div d^-1^)^-1^ for palmitic (C_16:0_), and 32‰ (div d^-1^)^-1^ for stearic acid (C_18:0_). (B) Fractionation factors (α) decreased, indicating greater ^2^H/^1^H fractionation between alkenones and extracellular water, as growth rates increased by 38‰ (div d^-1^)^-1^ for C_37:2_, and 25‰ (div d^-1^)^-1^ for C_37:3_, 24‰ (div d^-1^)^-1^ for C_38:2_, and 33‰ (div d^-1^)^-1^ for C_38:3_.

Fractionation factors (α) generally decreased, indicating greater ^2^H/^1^H fractionation between lipids and extracellular water, as growth rates increased for all lipids. The magnitude of the increase in fractionation as a function of growth rate was between 52 and 79‰ (div d^-1^)^-1^ for myristic (C_14:0_), palmitic (C_16:0_), and stearic (C_18:0_) fatty acids (Fig 3A), 44‰ (div d^-1^)^-1^ for brassicasterol (Fig 3A), and between 24 and 38‰ (div d^-1^)^-1^ for the four alkenones (Fig 3B).

The alkenone unsaturation index, U^k’^_37_, values in the *E*. *huxleyi* chemostat cultures were between 0.675 and 0.733 and did not vary systematically with growth rate (Table 2). These U^k’^_37_ values correspond to water temperatures of 18.7°C to 20.4°C using the Prahl et al. (1988) temperature calibration [42], close to the growth temperature of 20°C.

### *T. pseudonana*

**Fig 4:**
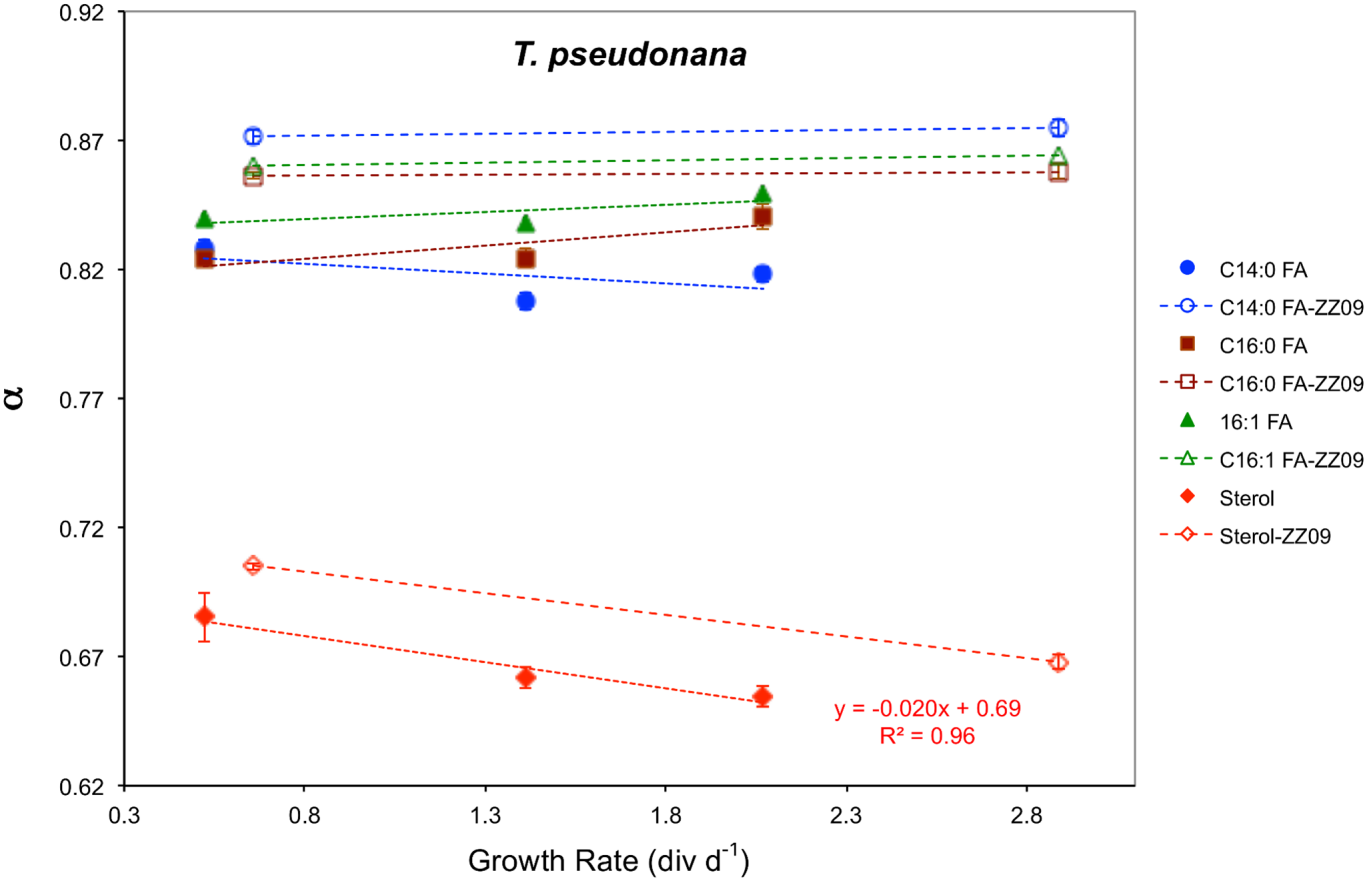
Hydrogen isotope fractionation in 24-methyl-cholesta-5,24(28)-dien-3β-ol and three fatty acids as a function of growth rate in *T*. *pseudonana* chemostat cultures. Open symbols are results reported in [37]. Fractionation factors (α) decreased in the sterol, indicating greater ^2^H/^1^H fractionation between lipids and extracellular water, as growth rates increased by 20‰ (div d^-1^)^-1^, and nearly constant in C_14:0_, C_16:0_ and C_16:1_ fatty acids.

The marine centric diatom *Thalassiosira pseudonana* (CCMP 1335) was grown in continuous cultures at 0.52, 1.41, and 2.07 div d^-1^ (Table 1). Concentrations of lipids were between 9.7 fg cell^-1^ and 17 fg cell^-1^ for 24-methyl-cholesta-5,24(28)-dien-3β-ol, and between 1.3 fg cell^-1^ and 110 fg cell^-1^ for C_14:0_, C_16:0_, C_16:1_, C_18:0_, C_18:1_ fatty acids (Fig 2 and Table 4). δ^2^H values were between -394‰ and -366‰, resulting in fractionation factors between 0.655 and 0.685 in the sterol, and between -252‰ and -214‰, resulting in fractionation factors between 0.808 and 0.849 in the fatty acids (Table 3 and Fig 4).

**Table 4:** Lipid concentrations in *T*. *pseudonana* chemostat cultures.

		Fatty Acids (fg cell^-1^)				
Growth Rate (div d^-1^)	Sterol*(fg cell^-1^)	C_14:0_	C_16:1_	C_16:0_	C_18:1_	C_18:0_
0.52	14.1	5.16	14.0	48.4	1.27	3.60
1.41	16.8	89.6	77.7	145	5.39	4.83
2.07	9.68	50.5	55.3	112	3.54	4.92

* 24-methyl-cholesta-5,24(28)-dien-3β-ol.

Concentrations of lipids per cell in *T*.*pseudonana* chemostat cultures in fg (10^−15^ g) per cell.

## Discussion

### Lipid concentration variations as a function of growth rate

The increase in sterol and alkenone concentrations in *E*. *huxleyi* (Fig 1A) as growth rate decreased is consistent with many studies showing that phytoplankton respond to nitrogen limitation by accumulating lipids [43–47]. Several studies have further demonstrated that alkenone concentrations increase under N limitation in *E*. *huxleyi* [48–51] and the related haptophyte species *Gephyrocapsa oceanica* [50] and *Isochrysis galbana* [49,52]. All of these studies were conducted with batch cultures, and N limitation was usually associated with the post-exponential or stationary phase of growth, making direct comparison with our results from continuous cultures of exponentially growing cells difficult. Nevertheless, the systematic increase in alkenone concentrations in haptophyte cells that are limited by nitrogen, regardless of growth phase or experimental treatment, implies a robust physiological response.

Some fatty acid concentrations decreased (C_14:0_, C_16:0_), while others increased (C1_8:1_) in *E*. *huxleyi* (Fig 1B) as growth rate decreased. While there may have been a decline in fatty acid content of cells in *T*. *pseudonana* (Fig 2) as growth rate decreased the data are inconclusive with just 3 data points.

### Hydrogen isotope variations as a function of growth rate in *E*. *huxleyi*

As *E*. *huxleyi* growth rates increased from 0.2 to 1 div d^-1^ the apparent lipid-water ^2^H/^1^H fractionation increased (i.e., α decreased) by 52 to 79 ‰ (div d^-1^)^-1^ in fatty acids, 44 ‰ (div d^-1^)^-1^ in brassicasterol (Fig 3A) and 24 to 38 ‰ (div d^-1^)^-1^ in alkenones (Fig 3B). We put forth two hypotheses to explain this response. The first calls upon an increase in the fraction of NADPH used for lipid synthesis from the oxidative pentose phosphate (OPP) pathway, relative to that from the light reactions of photosynthesis. The second hypothesis attributes the high sensitivity of hydrogen isotope fractionation in algal lipids to growth rate to increased cellular demand for energy (adenosine triphosphate, ATP) and reductant (NADPH) at higher growth rates and the exchange of hydrogen between sugars and intracellular water.

#### Varying sources of NADPH

By 1981 it was recognized that the primary source of deuterium depletion in microalgal biomass is the hydride derived from NADPH during biosynthetic reactions [28,29,31–33]. H^-^ from NADPH produced photosynthetically by ferredoxin-NADP+ reductase in Photosystem I (PS1) is estimated to have a δ^2^H value about 600‰ lower than that of the water from which it was derived [53]. A fractionation factor (α) of 0.4 for hydride produced by photosynthetic oxidation of water is plausible according to Schmidt et al. (2003), in light of the theoretical α value of 0.36 derived from the dissociation constants for ^2^H_2_O relative to H_2_O, and the experimentally determined ^2^H/^1^H fractionation for water fission [29].

The central role that NADPH plays in imparting ^2^H-depletion to biomolecules appears to be independent of the Domain of Life or metabolism (e.g., heterotrophic, photoautotrophic, chemoautotrophic) of the organism [31]. Culture studies with photoautotrophic eukaryotic unicellular phytoplankton [9,21,28], photoautotrophic, photoheterotrophic and heterotrophic C_3_ plants [32], photoheterotrophic unicellular eukaryotes [54], heterotrophic, chemoautotrophic and photoautotrophic prokaryotes [31], and heterotrophic archaea [55] all conclude that hydride derived from NADPH is the principle source of ^2^H-depletion in lipids relative to environmental water.

Because NADPH and the associated reductant nicotinamide adenine dinucleotide (NADH) can be produced via multiple pathways, including the light reactions of photosynthesis (in photoautotrophs), the oxidative pentose phosphate (OPP) pathway, the tricarboxylic acid (TCA) cycle, glycolysis, and the glyoxylate cycle, the relative contributions of reductant to a biomolecule from these various pathways is likely to be an important source of H isotopic variation between different lipids in the same cell and between the same lipid in different cells [31,55]. Changing sources of NADPH within a cell in response to environmental conditions and/or metabolic state can therefore be expected to give rise to differing magnitudes of ^2^H-depletion in lipids.

NADPH derived from processes other than PS1 is expected to be enriched in ^2^H relative to that produced photosynthetically by ferredoxin-NADP+ reductase in PS1 [29], because it acquires hydride from metabolites rather than photooxidized water. For example, hexoses in the cytosol provide the hydride from which NADPH is produced in the cytosolic OPP pathway. Hydrogen in those hexoses derives ultimately from both intracellular water and NADPH. Assuming half of that hydrogen comes from each, as is the case for glyceraldehyde 3-phosphate (GAP) produced in the Calvin cycle, GAP and monosaccharides synthesized from it might be expected to have a δ^2^H value of approximately -300‰ (assuming δ^2^H_NADPH/PS1_ = -600‰ and δ^2^H_water_ = 0‰). Normal isotope effects for glucose-6-phosphate dehydrogenase and 6-phosphogluconate, the enzymes catalyzing the reduction of NADP^+^ in the OPP pathway might result in NADPH with a δ^2^H value less than that on the one hand, but hydrogen exchange reactions between water and saccharides prior to and during the OPP pathway may increase their δ^2^H value. The latter effect could be exacerbated by the fact that deuterium is preferentially enriched in carbohydrates during hydrogen exchange with water [33]. For these reasons the apparent fractionation factor for non-photosynthetic NADPH is expected to be higher (less ^2^H/^1^H fractionation) than for photosynthetically produced NADPH, closer to 0.75 [29] than the 0.4 proposed for photosynthetic NADPH.

A secondary source of ^2^H-enrichment of hydride from both PS1 and the OPP pathway may be hydrogen exchange between intracellular water and NADPH-derived H^-^ during its transfer to lipids via flavoproteins [29,31]. Hydride can be directly transferred to lipids from NADPH or it can first be transferred to a flavoprotein, and then to the lipid. Once associated with the flavin ring H^-^ can exchange with water [56], resulting in ^2^H-enrichment assuming a normal isotope effect. The extent to which hydrogen exchange during hydride transfer via flavoproteins influences lipid δ^2^H values will depend on the enzymes involved with lipid and precursor synthesis since some enzymes are flavin-free, the type of lipid, and the species of phytoplankton. Since virtually nothing is known about the extent to which H^-^ transfer via flavoproteins is likely to influence lipid δ^2^H values in phytoplankton we neglect this process in the following discussion and propose simply that it may act to decrease the δ^2^H difference between lipids and intracellular water.

**Fig 5:**
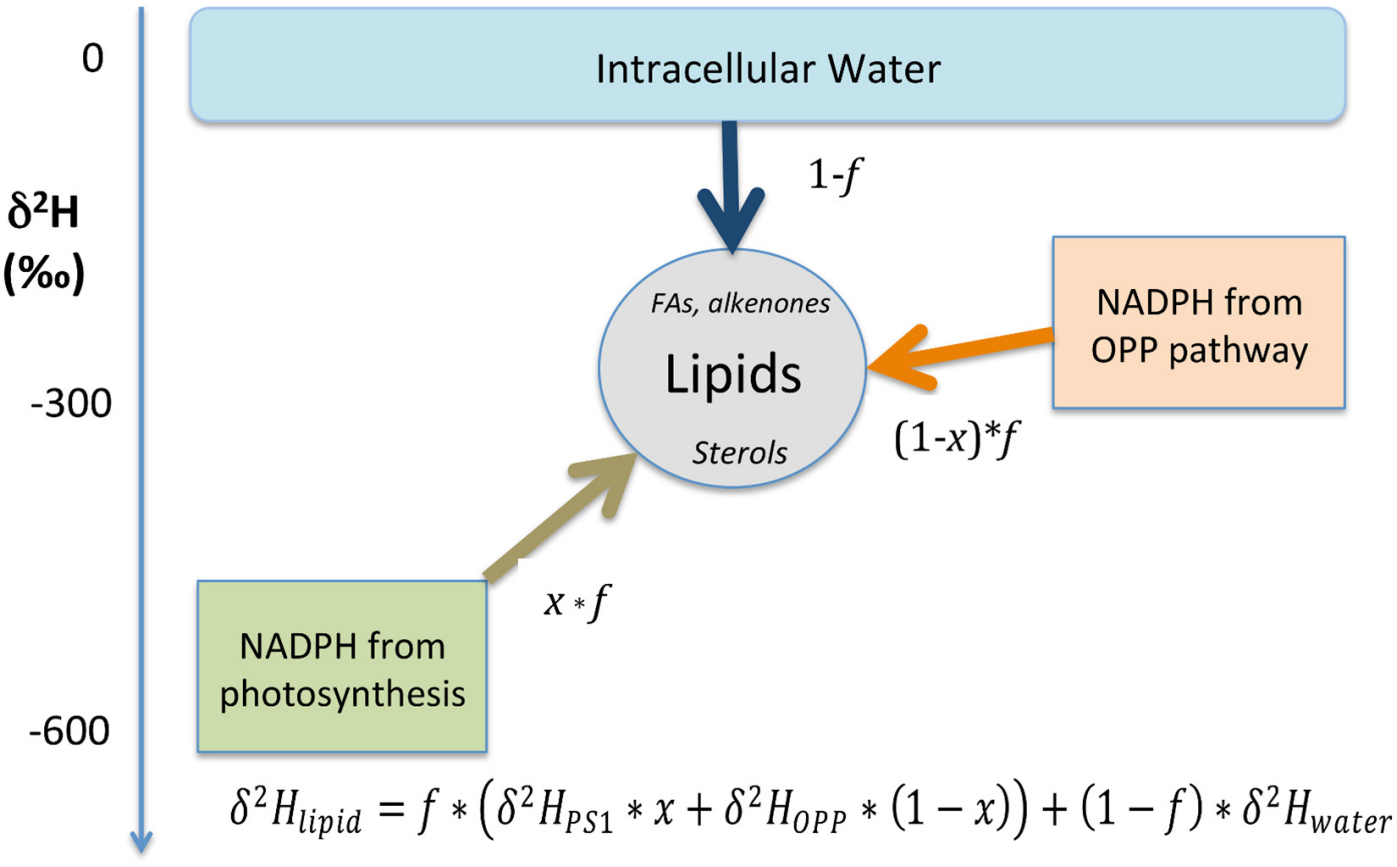
Model of hydrogen isotopic relationships giving rise to observed δ^2^H values of lipids in *E*. *huxleyi* cells. *f* is the fraction of hydrogen in lipids that comes from NADPH, 1-*f* is the fraction of hydrogen in lipids that comes from water, *x* is the fraction of hydrogen in lipids derived from PS1 of photosynthesis, *1-x* is the fraction of hydrogen in lipids derived from the OPP pathway.

A simple mass balance model demonstrates the sensitivity of algal lipid δ^2^H values to changes in the relative proportion of H^-^ derived from photosynthetic and non-photosynthetic NADPH (Fig 5). Here we assume that all of the hydrogen in lipids (fatty acids, alkenones and sterols in the case of *E*. *huxleyi*) derives from three sources: photosynthetic and non-photosynthetic NADPH and intracellular water. At the steady state condition represented by the continuous cultures the following mass balance yields the δ^2^H value of lipids:

δH2lipid=f*(δH2NADPH/PS1*x+δH2NADPH/opp(1−x))+(1−f)*δH2water (1)

where *f* is the fraction of hydrogen in lipids that comes from NADPH and *x* is the fraction of that NADPH produced in PS1 of photosynthesis. According to this model, when apparent fractionation factors for photosynthetic (0.4) and non-photosynthetic (0.75) NADPH are used, the δ^2^H value of intracellular water is assumed to be the same as that for extracellular water (0‰), and 50% of the hydrogen in lipids is assumed to come from each NADPH and intracellular water, δ^2^H_lipid_ is -213‰ and the fractionation factor (α) for lipids is 0.787. This is close to the average for acetogenic lipids (i.e., alkenones and fatty acids) in *E*. *huxleyi* cultures (Table 3, Fig 3).

Based on this mass balance model we propose four relationships that can account for (i) the universal ^2^H-depletion in the lipids of phytoplankton relative to environmental water, (ii) the increase in ^2^H-depletion of lipids as growth rate increases, the (iii) ^2^H-depletion in isoprenoid lipids relative to acetogenic lipids, and (iv) the ^2^H-depletion in lipids relative to carbohydrates and protein:

δH2H2Oi > δH2NADPH/opp > δH2NADPH/PS1 (2)

x∝growth rate (3)

$$f_{isoprenoid}\;>\;f_{acetogenic}$$ (4)

$$f_{lipids}\;>\;f_{carbohydrate+protein}$$ (5)

The first relationship (Eq 2) states that the δ^2^H value of intracellular water is greater than that of hydride from NADPH produced via the OPP pathway, which in turn is greater than the δ^2^H value of H^-^ produced in PS1, as described by Schmidt et al. (2003) [29]. The second (Eq 3) states that the relative proportion of NADPH from PS1 and the OPP pathway is proportional to growth rate, justified below, and explaining why α values decrease as growth rate increases (Fig 3). The third (Eq 4) states that there is a greater proportion of hydrogen derived from NADPH relative to water in isoprenoid lipids as compared to acetogenic lipids, explaining their greater ^2^H-depletion. The fourth (Eq 5) states that there is a greater proportion of hydrogen derived from NADPH relative to water in lipids as compared to other cellular biomass (i.e., carbohydrates plus proteins), explaining their greater ^2^H-depletion, as first observed by Estep et al. (1980) [21].

**Fig 6:**
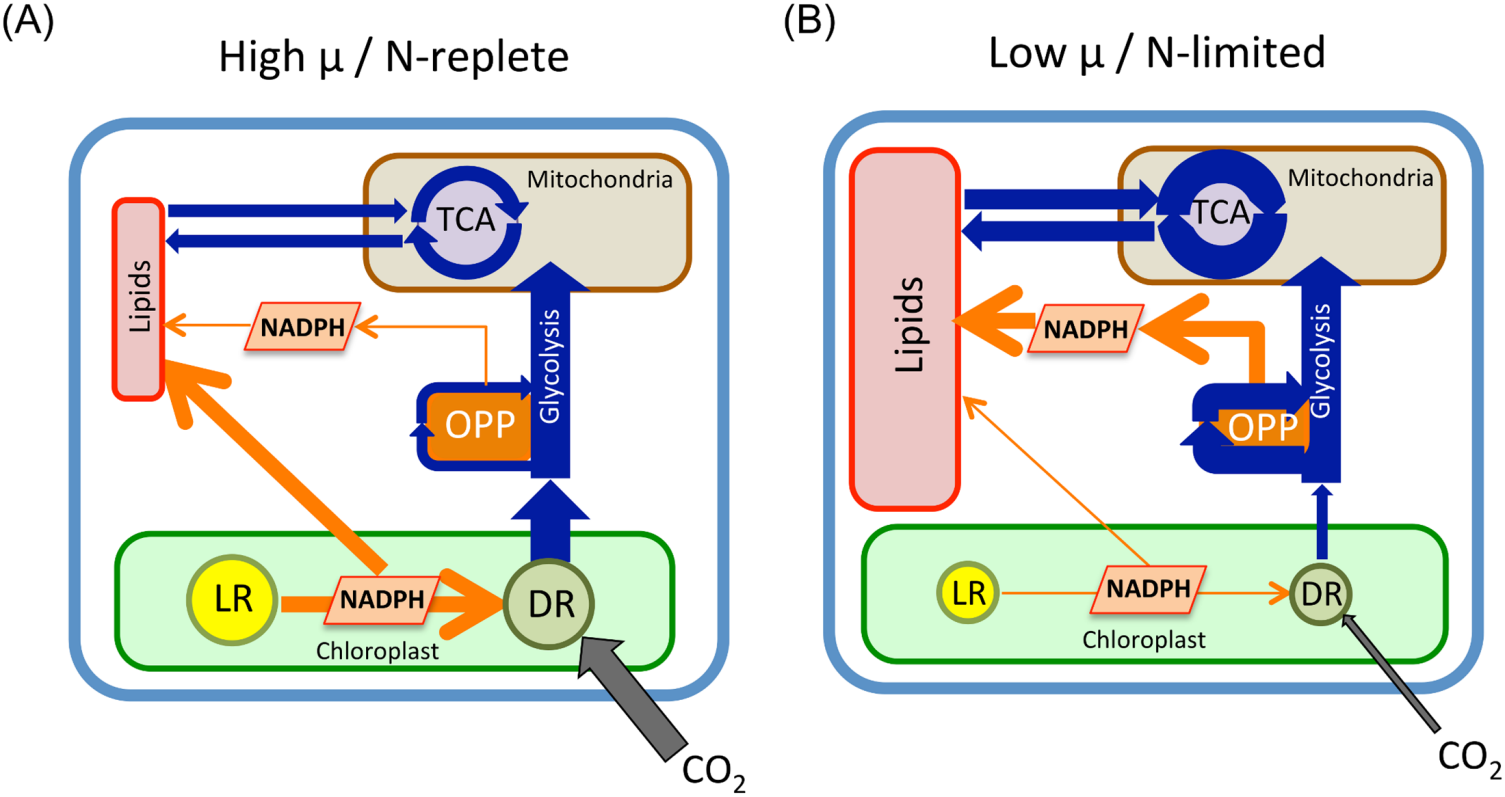
Proposed metabolic differences in *E*. *huxleyi* cells growing at different rates. The two represented regimes are: (A) high rates of growth and/or in N-replete conditions, and (B) low rates of growth and/or in N-limited conditions (after [74], Fig 3). LR = Light Reactions of photosynthesis. DR = Dark Reactions (Calvin Cycle). OPP = Oxidative Pentose Phosphate pathway. TCA = Tricarboxylic Acid Cycle.

The utility of this model to explain the decrease in α as growth rate increased in *E*. *huxleyi* rests on the assumption that the proportion of H^-^ from OPP increased at the expense of H^-^ from PS1 as growth rate decreased. While we have no direct evidence for this assertion, a substantial body of literature on the response of phytoplankton cells to nitrogen limitation supports its plausibility. According to Hockin et al. (2012) “The down-regulation of photosynthesis is a universal response to nitrogen starvation among photosynthetic eukaryotes” [57]. Photochemical energy conversion efficiency decreases and genes associated with photosynthesis and carbon fixation are down-regulated [57–59]. In *E*. *huxleyi* Rokitta et al. (2014) concluded that “the photosynthetic light reactions were strongly decreased” under N-limitation based on the down regulation of genes associated with plastidic ATP and chlorophyll synthesis [60]. At the same time genes associated with the OPP pathway and TCA cycle are up-regulated in N-limited cells to increase the efficiency of intracellular nitrogen assimilation [57–59]. A decrease in photosynthesis combined with an up-regulation of OPP and TCA genes in response to N-limitation is likely to cause a shift in the proportion of NADPH derived from those processes, such that relatively-less-^2^H -depleted NADPH from OPP+TCA is produced at the expense of more-highly-^2^H -depleted NADPH from photosynthesis as N-limited growth rate decreases. A schematic representation of these metabolic changes is shown in Fig 6. At low rates of growth and/or under N-limited conditions genes associated with photosynthesis and carbon fixation are down-regulated, indicated in Fig 6B by smaller compartments for the light (LR) and dark (DR) reactions of photosynthesis, and a smaller flux of CO_2_ to the DR. At the same time genes associated with the OPP pathway and TCA cycle are up-regulated and cellular production of lipids is high. We propose that this constellation of metabolic activity results in a relatively larger proportion of NADPH used for lipid synthesis coming from OPP compared to PS1, and therefore a relatively higher δ^2^H value of those lipids. Conversely, we propose that at high rates of growth and/or under N-replete conditions genes associated with photosynthesis and carbon fixation are up-regulated, resulting in a relatively large flux of reductant from photosynthesis as compared to the OPP pathway for the synthesis of lipids (Fig 6A).

**Fig 7:**
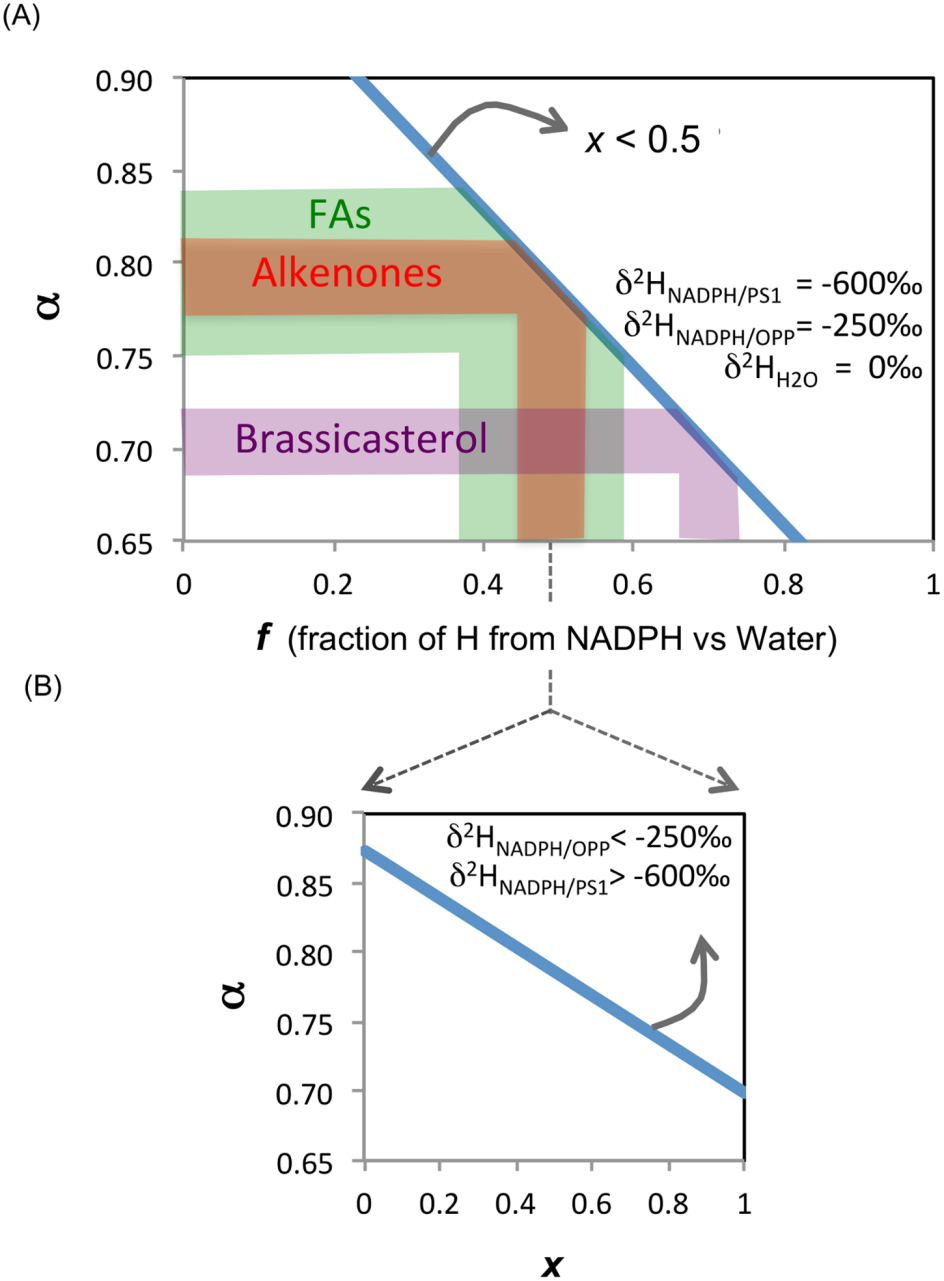
Sensitivity of the ^2^H/^1^H fractionation factor, α, to intracellular hydrogen source. The fractionation factor, α, can respond to both (A) *f*, the fraction of lipid hydrogen derived from NADPH versus intracellular water, and (B) *x* the fraction of NADPH-derived hydrogen in lipids that comes from photosynthesis as opposed to the OPP pathway. δ^2^H values of NADPH/PS1 and NADPH/OPP are set at -600‰ and -250‰, respectively. Intracellular water δ^2^H is set at 0‰. The shaded areas in (A) indicate the range of α values measured for 3 lipid classes (fatty acids, FA-green; alkenones-red; brassicasterol-purple) in our *E*. *huxleyi* continuous cultures (Table 3). The slope of the relationship would increase if less than half of the NADPH-derived hydrogen in lipids came from photosynthesis (i.e., *x* < 0.5 In (B) it is assumed that half of the hydrogen in lipids is from NADPH and half from water (i.e., *f* = 0.5). A greater fraction of NADPH from photosynthesis (higher *x*) results in lower α values since photosynthetically produced hydride is ^2^H -depleted relative to NADPH produced via OPP in the cytosol. In (B) the sensitivity of α to changes in *x* decreases if either δ^2^H _NADPH/PS1_ > -600‰ or δ^2^H _NADPH/OPP_ < -250‰.

Fig 7 illustrates the sensitivity of α to changes in *f*, the fraction of lipid hydrogen derived from NADPH versus intracellular water, and *x*, the fraction of NADPH-derived hydrogen in lipids that comes from PS1 as opposed to the OPP pathway. ^2^H-depletion increases (α decreases) as both *f* (Fig 7A) and *x* (Fig 7B) increase. The sensitivity of α to *f* increases (i.e., the slope in Fig 7A increase) as the proportion of NADPH from PS1 decreases. The sensitivity of α to *x* decreases (i.e., the slope in Fig 7B decrease) if either δ^2^H_NADPH/PS1_ increases or δ^2^H _NADPH/OPP_ decreases. When fractionation factors for NADPH from PS1 and OPP are set at 0.4 and 0.75, respectively, δ^2^H_H2Oi_ is set at 0‰, and half of the NADPH used in lipid synthesis comes from PS1, the model predicts that roughly 40% to 60% of hydrogen in fatty acids, 45% to 55% of hydrogen in alkenones, and 65% to 75% of hydrogen in brassicasterol comes from NADPH, with the remainder coming from water (Fig 7A). These estimates are consistent with the assessment by [31] that about 50% of the hydrogen in fatty acids comes from NADPH, and 25% each from water and acetyl-CoA.

#### ^2^H-depletion of intracellular water at high growth rates

A second mechanism by which ^2^H/^1^H fractionation in lipids could increase with growth rate is a lowering of δ^2^H_H2Oi_ from more rapid hydrogen exchange between relatively ^2^H-enriched cell water and relatively ^2^H-depleted organic hydrogen at higher growth rates. It has been shown that hydrogen from intracellular water is rapidly and extensively exchanged with certain hydrogen atoms in biomolecules, such as those bound to O, P and N [29,30,32]. Even hydrogen atoms that are bound to C can readily exchange in the aqueous medium of the cell when they occur adjacent to certain functional groups, such as ketones and aldehydes, via keto-enol tautomerism. The rates of non-enzymatic hydrogen exchange reactions are often much greater than for enzyme-mediated reactions [30].

In a series of ^14^C labeling experiments with the green alga *Dunaliella tertiolecta*, Halsey et al. (2011) showed that the turnover of polysaccharides was eight times faster in cells growing at 1.7 div d^-1^ than in cells growing at 0.17 div d^-1^ in the four hours following the introduction of DI^14^C [61]. This was attributed to rapid catabolism of carbohydrates in the TCA cycle and the OPP pathway in fast-growing cells. Based on these results, and the well-established relationship between metabolic rates and growth rates in plants and animals [62,63] it is reasonable to assume that the metabolic rates in the *E*. *huxleyi* cultures co-varied with growth rate.

Whether δ^2^H_H2Oi_ scales with growth rate will then depend on whether the rate of hydrogen exchange between organic hydrogen and intracellular water scales with the metabolic rate. If so, then fast-growing cells ought to have δ^2^H_H2Oi_ values that are lower than slow-growing cells as the H-exchange process transfers ^2^H-depleted hydrogen to the water. Experimental evidence for greater H-exchange at higher growth rates in prokaryotic and mammalian cells was provided by [34,35]. They demonstrated that the fraction of hydrogen in intracellular water that had been metabolically processed was about 50% in *E*. *coli* and rat fibroblast cells in the exponential phase of growth as compared to about 25% in cells at the stationary phase of growth [34,35]. Whether photoautotrophic cells such as *E*. *huxleyi* exhibit similar rates of H-exchange is unknown.

Any tendency to lower δ^2^H_H2Oi_ via H-exchange with organic hydrogen would be countered by the ^2^H-enrichment of cell (specifically, plastidic) water that presumably accompanies water oxidation in PS1 and by the exchange of intra- and extra-cellular water. On the other hand, the exchange of hydrogen between carbohydrates and water enriches the carbohydrate in ^2^H, which would drive δ^2^H_H2Oi_ lower. The net effect of H-exchange between organic hydrogen and intracellular water on δ^2^H_H2Oi_, and whether a lowering of δ^2^H_H2Oi_ as growth rate increases is a viable mechanism for decreasing α as growth rate increases remain open questions. Future experiments ought to address these questions by measuring δ^2^H_H2Oi_.

### Generality of growth rate influence on ^2^H/^1^H fractionation

**Fig 8:**
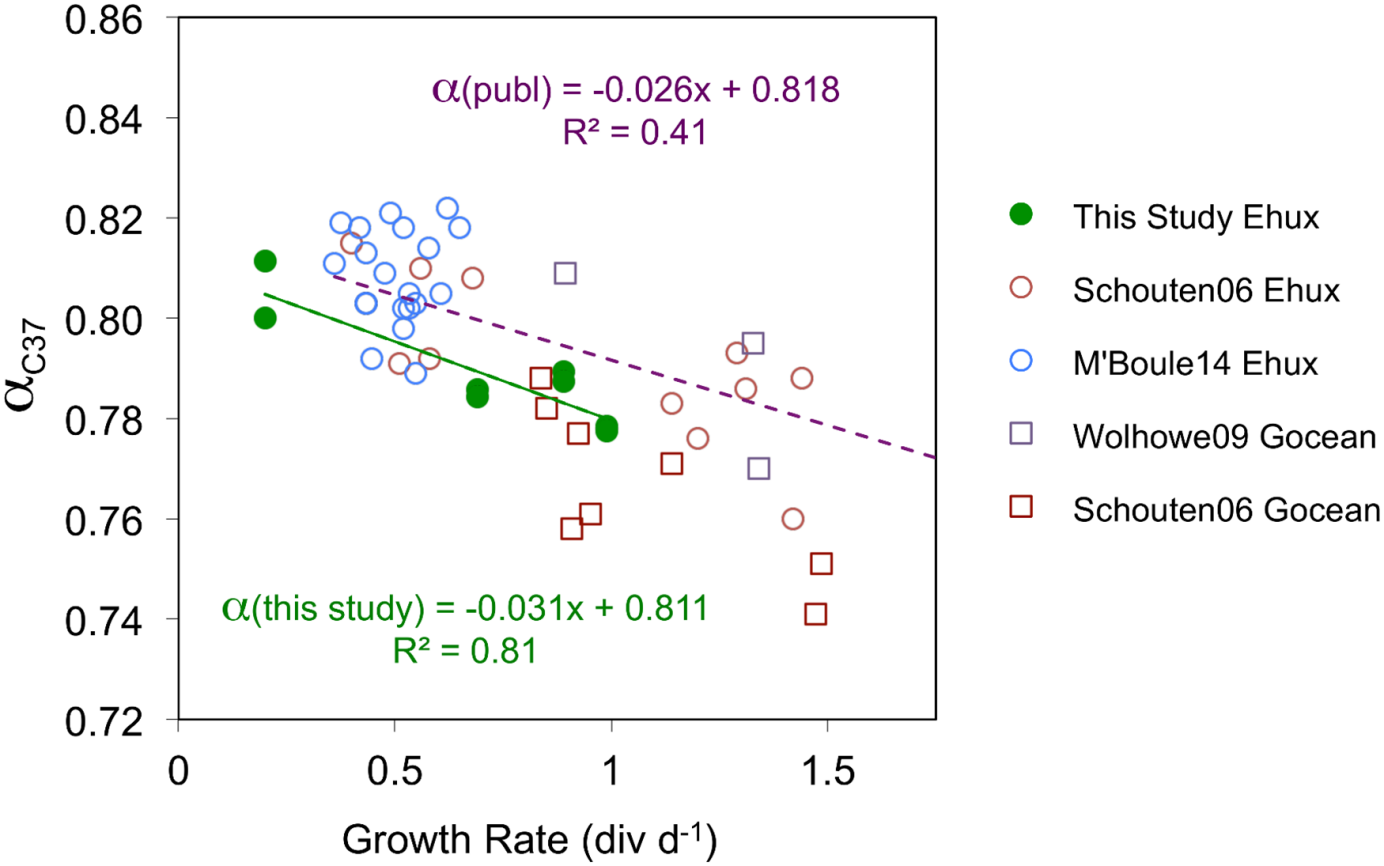
Fractionation factor for C_37_ alkenones as a function of growth rate in *E*. *huxleyi* and *G*. *oceanica* cultures. The presented results are from the following sources: *E*. *huxleyi* data: continuous cultures from this study (solid green circles), batch cultures from [10] (open brown circles) and [36] (open blue circles). *G*. *oceanica* data: batch cultures from [10] (open brown squares) and [23] (open purple squares). All data are from C_37_ methyl alkenones. The C_37:2_ and C_37:3_ alkenones were measured and plotted separately in this study, whereas they were combined and measured together in [10,23,36].

Since the optimal, or “Redfield” ratio of N:P is 16 we assume that all of the cultures grown in N2L media that had N:P ratios of 1 to 1.5 (*E*. *huxleyi* 0.2 and 0.69 div d^-1^), 2.4 (*T*. *pseudonana* 0.66 div d^-1^ [37]) or 5 (*T*. *pseudonana* 0.52, 1.41 and 2.07 div d^-1^) were N-limited and that the cultures grown in NR media that had molar N:P ratios of 24 (*E*. *huxleyi* 0.89 and 0.99 div d^-1^) or 16 (*T*. *pseudonana* 2.89 div d^-1^ [37]) were not N-limited [64,65]. It is possible therefore that growth rate itself, be it modulated by substrate limitation, temperature or salinity, rather than N-limitation, is fundamentally linked to ^2^H/^1^H fractionation. Support for this comes from published batch culture experiments in which growth rates of the coccolithophorids *E*. *huxleyi* and *Gephyrocapsa oceanica* were inferred to have changed as salinity and temperature were altered [10,36]. As growth rate increased in their *E*. *huxleyi* cultures the ^2^H/^1^H fractionation between C_37_ alkenones and growth water increased by 26‰ (div d^-1^)^-1^, within 20% of the increase observed in our *E*. *huxleyi* chemostats (31‰ (div d^-1^)^-1^) (Fig 8). This suggests that our 3-endmember model of hydrogen isotopes in phytoplankton lipids (Fig 5) and the metabolic differences in *E*. *huxleyi* cells at high (Fig 6A) and low (Fig 6B) growth rates may be equally valid whether the growth rate differences are induced by N-limitation, temperature or salinity.

### Growth rate influence on H isotope variations in *T*. *pseudonana* lipids

The results of the *T*. *pseudonana* chemostat experiments are not as conclusive as those for *E*. *huxleyi* owing to the fact that two of the five *T*. *pseudonana* chemostats were conducted using a different protocol (see [37]) than the three chemostats conducted as part of this study, apparently resulting in α values some 0.02 to 0.05 higher (Fig 4). Trace metal concentrations in particular were 16 to 44 times higher in the three chemostats conducted for this study than in the two chemostats reported in [37] except for molybdenum which was 25% of the concentration used in the previous study (Table A in S1 Appendix). Additionally, chemostat cultures grown for this study had 10% the EDTA concentration as those in [37]. The very different per cell sterol and C_16:0_ FA concentrations in the [37] cultures as compared to the three new cultures (Fig 2) is a good indication that the metabolic state of *T*. *pseudonana* cells in the two studies was different, irrespective of growth rate. If the lower trace metal concentrations in the earlier study were responsible for their higher α values, one possibility is that a smaller proportion of NADPH from PS1 relative to OPP is available for lipid synthesis when one or more trace metals are limiting.

Notwithstanding these differences in the two studies there appears to be a trend toward lower α values and greater ^2^H/^1^H fractionation at higher growth rates in the sterol (24-methyl-cholesta-5,24(28)-dien-3β-ol) (Fig 4), similar to the trend observed in *E*. *huxleyi* cultures (Fig 3). As in all *E*. *huxleyi* lipids, greater ^2^H/^1^H fractionation in the *T*. *pseudonana* sterol at higher growth rates can be explained by a proportional increase in the fraction of NADPH derived from PS1 relative to OPP as growth rate increases.

The seemingly anomalous finding is the lack of any clear trend in fatty acid α values as a function of growth rate in either of the two sets of *T*. *pseudonana* cultures, supporting the inference from [37] that ^2^H/^1^H fractionation in fatty acids from *T*. *pseudonana* is insensitive to growth rate (Fig 4). Based on the simple model we have proposed to explain hydrogen isotopic relationships of lipids in phytoplankton and their response to growth rate changes (Eqs 1–5, Fig 5) this lack of sensitivity of α to growth rate could be explained by an increasing fraction of hydrogen in fatty acids derived from (isotopically enriched) intracellular water (*f*) as growth rate increases that offsets the greater fraction of (isotopically depleted) NADPH from photosynthesis (*x*) that the model assumes. Evidence in support of this possibility comes from the overall decrease in cellular metabolism when *T*. *pseudonana* is deprived of nitrogen [59] coupled with the observations by [34,35] that the flux of hydrogen between intracellular water and metabolites is greater in exponentially growing cells than in stationary cells.

At this point it can be concluded that the mechanism put forth to explain increasing ^2^H/^1^H fractionation at higher growth rates in the *T*. *pseudonana* sterol by [37] is unlikely to be correct. They attributed greater fractionation at higher growth rates to the transfer of ^2^H-depleted isopentenyl diphosphate (IPP) from the plastid (where it is synthesized in the 1-deoxyxylulose 5-phosphate/2-*C*- methyl-D-erythritol 4-phospate (DOXP/MEP) pathway) to the cytosol to feed into the acetate mevalonic acid (MVA) pathway of sterol synthesis. This no longer seems viable because the sterol in *T*. *pseudonana* responds just like the fatty acids, alkenones and sterol in *E*. *huxleyi*, with a decrease in α as growth rate increases (Fig 3), and IPP is not involved in acetogenic lipid synthesis.

### Implications for paleoclimate reconstructions

Owing to their source specificity and excellent preservation in the geologic record alkenone δ^2^H values are increasingly being used to reconstruct hydrologic conditions in paleoclimatology [8,14–16,19,20]. Our observation that δ^2^H values of alkenones decrease by approximately 30‰ (div d^-1^)^-1^ has the potential to complicate their interpretation as hydroclimate indicators. Relatively small growth rate changes of 0.1 to 0.2 div d^-1^ would be expected to cause alkenone δ^2^H differences of 3‰ to 6‰, which is approximately the analytical precision of the analysis. Larger (alkenone-producing) coccolithophorid growth-rate differences, either at one location over time, between locations, or between a location and the calibration set have the potential to influence results, especially if the differences are systematic.

For example, within the euphotic zone of the subpolar North Pacific Ocean the growth rate of alkenone-producing coccolithophorids varied between 0.9 div d^-1^ at 10 m and 0.1 div d^-1^ at 70 m in [66]. Most of that decline was attributed to decreasing light intensity [66]. If growth rate controlled by light affects ^2^H/^1^H fractionation in phytoplankton the same way other growth parameters do (e.g., N-limitation, temperature and salinity), alkenones produced at 10 m should have had a δ^2^H value about 25‰ less than those produced at 70 m. For comparison, that would be the same signal that would be expected from a 10 to 20 ppt change in salinity [22,36]. Thus, if alkenone δ^2^H values were used to reconstruct salinity changes from a sediment core at the subpolar location in [66] there could be as much as a 10–20 ppt uncertainty in reconstructed salinity depending on the depth at which the alkenones in the sediment were initially synthesized.

#### No influence of growth rate on alkenone unsaturation ratios

The relative abundances of the di- and tri-unsaturated C_37_ methyl alkenones, the U^k’^_37_ ratio [67], showed no systematic change as a function of growth rate (Table 2). This result adds to the substantial body of literature supporting the robustness of the alkenone thermometer under a very wide range of environmental conditions [68–73]. On the other hand, [23] reported that batch cultures of both *G*. *oceanica* and *E*. *huxleyi* had systematically lower U^k’^_37_ values at the stationary phase and “late-log” phase of growth compared to the exponential and “mid-log” phase of growth. Our continuous culture results do not conflict with those results as the growth phase under the two types of culture systems are distinctly different, with cells in the chemostats undergoing perpetual exponential growth. Nevertheless, the comparison does support the inference that the growth phase of cells that produce the alkenones ultimately buried in sediments could influence their U^k’^_37_ value, independent of temperature. The use of core-top calibrations of the alkenone thermometer (e.g., [70]), rather than those from culture or suspended particles, ought to mitigate this effect. The fact that global core-top calibrations are virtually identical to those from culture (compare [70] to [42]) further implies that the effect of growth phase on U^k’^_37_ is likely to be small compared to that of temperature.

## Conclusion

Continuous culture experiments with the coccolithophorid *Emiliania huxleyi* revealed that hydrogen isotope fractionation in both isoprenoid and acetogenic lipids increases as growth rate increases. δ^2^H values decreased by 24 to 38‰ (div d^-1^)^-1^ in di-and tri-unsaturated C_37_ and C_38_ alkenones, 44‰ (div d^-1^)^-1^ in brassicasterol, and 32 to 79‰ (div d^-1^)^-1^ in myristic acid (C_14:0_), palmitic (C_16:0_), and stearic acid (C_18:0_). A simple 3-endmember mixing model in which hydrogen in lipids comes from NADPH produced in Photosystem 1 of photosynthesis with α ~ 0.4, NADPH produced by the cytosolic oxidative pentose phosphate pathway with α ~ 0.75, and intracellular water with α ~ 0 is used to explain these results. We propose that the fraction of NADPH from PS1 increases as growth rate increases, resulting in a lowering of α. Support for this mechanism comes from transcriptomic studies in which genes associated with photosynthesis and carbon fixation are down-regulated while those associated with OPP are up-regulated in N-limited cells. We hypothesize that the resulting constellation of metabolic changes causes a shift in the proportion of NADPH derived from PS1 and the OPP pathway, such that relatively less ^2^H-depleted NADPH from OPP is produced at the expense of more highly ^2^H-depleted NADPH from PS1 as N-limited growth rate decreases. The model can also account for the observations that isoprenoid lipids are depleted in ^2^H relative to acetogenic lipids and the δ^2^H values of lipids are lower than those of proteins and carbohydrates if the fraction of hydrogen from intracellular relative to NADPH is greater in acetogenic lipids, proteins and carbohydrates.

While the model successfully explains the increase in ^2^H/^1^H fractionation in the sterol 24-methyl-cholesta-5,24(28)-dien-3β-ol from *T*. *pseudonana* chemostat cultures as growth rate increases, an additional process must be invoked to explain the lack of sensitivity of ^2^H/^1^H fractionation in fatty acids to growth rate. An increase in the fraction of hydrogen in fatty acids that is derived from intracellular water at the expense of NADPH as growth rate increases is one possibility.

The ~30‰ decrease in the δ^2^H value of alkenones per unit (div d^-1^) increase of growth in our *E*. *huxleyi* chemostats is in line with published batch culture experiments showing a similar magnitude response. This needs to be considered when applying δ^2^H measurements of sedimentary alkenones in paleoclimate studies since any change in growth rate larger than about 0.15 div d^-1^ would cause a change in δ^2^H_lipid_ larger than the analytical error of the measurement, about 5‰.

## Supporting Information

S1 Appendix Growth media comparison for *T*. *pseudonana* continuous cultures. (DOCX)

S2 Appendix Lipid extraction and class separation. (DOCX)

S3 Appendix HPLC-MS purification of alkenones and brassicasterol. (DOCX)

S4 Appendix Fatty acid and sterol derivatization. (DOCX)

S5 Appendix GC-FID and GC-MS analyses. (DOCX)

S6 Appendix GC-IRMS instrumentation for hydrogen isotope analysis of lipids. (DOCX)
