# Supplementary material for: The Influence of Growth Rate on 2H/1H Fractionation in Continuous Cultures of the Coccolithophorid Emiliania huxleyi and the Diatom Thalassiosira pseudonana
Source: PLoS One. 2015 Nov 17;10(11):e0141643. doi: 10.1371/journal.pone.0141643 (PMC4648508; doi:10.1371/journal.pone.0141643)
Supplement: S1 Appendix — (DOCX) [file pone.0141643.s001.docx]

**S1 Appendix. Growth media comparison for *T. pseudonana* continuous cultures**

A comparison of compositions of nitrogen-limiting (N2L) and nutrient replete (NL) growth media for the T. pseudonana cultures in this study with those of the referenced [37] study and standard f/2 [38-39] and Aquil [75] media is provided in Table 1. The vitamin concentrations in the feed media are consistent between the two studies referenced in the article regardless of the medium type. Most of the trace metal concentrations in the growth media of the current study (based on the f/2 recipe) are enriched relative to those in the previous [37] study (based on the Aquil medium). These enrichments range from a factor of 16 (CoCl_2_) to 44 (MnCl_2_). In contrast, the concentrations of Na_2_EDTA and Na_2_MoO_4_ are reduced by factors of 8.6 and 4.4, respectively. The macronutrient concentrations are specific to each study and reflect the N2L and NR nature of the various feed media.

75. Price NM, Harrison GI, Hering JG, Hudson RJ, Nirel PMV, Palenik B, Morel, FMM (1989) Preparation and chemistry of the artificial algal culture medium Aquil. Biological Oceanography 6: 443-461.

Table A Comparison of growth media for T. pseudonana cultures. See text for description.

| **Study or Reference Medium** | **Medium Type** | **Macronutrients** | | | | **Trace Metals** | | | | | | | | **Vitamins** | | |
| --- | --- | --- | --- | --- | --- | --- | --- | --- | --- | --- | --- | --- | --- | --- | --- | --- |
|  |  | **PO_4_ (µM)** | **Si(OH)_4_ (µM)** | **Nitrate (µM)** | **Molar N/P Ratio** | **FeCl_3_ (µM)** | **Na_2_EDTA (µM)** | **CuSO_4_ (nM)** | **Na_2_MoO_4_ (nM)** | **ZnSO_4_ (nM)** | **CoCl_2_ (nM)** | **MnCl_2_ (nM)** | **Na_2_SeO_3_ (nM)** | **Vitamin B12 (nM)** | **Biotin (nM)** | **Thiamine*HCl (uM)** |
| This study | N2L | 18.9 | 201 | 98.0 | 5.18 | 11.36 | 11.62 | 42.45 | 22.73 | 70.60 | 40.35 | 1010 | Not added | 0.443 | 2.46 | 0.304 |
| This study | NR | 37.8 | 106 | 891.2 | 23.56 | 11.36 | 11.62 | 42.45 | 22.73 | 70.60 | 40.35 | 1010 | Not added | 0.409 | 2.27 | 0.280 |
| Previous Study [37] | N2L | 17 | 175 | 40 | 2.35 | 0.45 | 100 | 0.997 | 100 | 4.00 | 2.50 | 23.0 | 10 | 0.411 | 2.05 | 0.296 |
| Previous Study [37] | NR | 17 | 175 | 270 | 15.9 | 0.45 | 100 | 0.997 | 100 | 4.00 | 2.50 | 23.0 | 10 | 0.411 | 2.05 | 0.296 |
| Aquil  Reference | – | 10 | 100 | 300 | 30.0 | 0.45 | 10 or 100 | 0.997 | 100 | 4.00 | 2.50 | 23.0 | 10 | 0.411 | 2.05 | 0.296 |
| f/2  Reference | – | 36.2 | 106 | 882 | 24.4 | 11.70 | 11.70 | 39.3 | 26.00 | 76.50 | 42.00 | 910 | – | 0.369 | 2.05 | 0.296 |
