# Supplementary material for: The Influence of Growth Rate on 2H/1H Fractionation in Continuous Cultures of the Coccolithophorid Emiliania huxleyi and the Diatom Thalassiosira pseudonana
Source: PLoS One. 2015 Nov 17;10(11):e0141643. doi: 10.1371/journal.pone.0141643 (PMC4648508; doi:10.1371/journal.pone.0141643)
Supplement: S2 Appendix — (DOCX) [file pone.0141643.s002.docx]

**S2 Appendix. Lipid extraction and class separation**

Lipids were extracted from the freeze-dried filter subsamples utilizing a Accelerated Solvent Extraction System 200 (Dionex ASE-200, Thermo Scientific, Sunnyvale, California, USA) and a 9:1 (v/v) mixture of dichloromethane:methanol as extraction solvent. The instrument method consisted of the following: Preheat, 2 min; Heat, 5 min; Static, 5 min; Flush, 100 % volume; Purge, 60 sec; Extraction Cycles, 3; Pressure, 1500 psi; Temperature, 100 °C. Full extraction of the lipids was assured by applying the method twice to each sample, i.e. once in each flow direction through the extraction cell. The two extracts were combined and solvent removed using a stream of pre-purified N_2_ and a water bath at 40 °C (TurboVap, Caliper, Hopkinton, Massachusetts, USA).

The lipids were separated into a non-polar (NP) and a polar, fatty-acid containing fraction (FA) using solid-phase extraction (SPE) on an aminopropyl column. The NP fraction contained the long-chain alkenones, alkenoates, and the primary sterol (24-methyl cholest-5,22-dien-3β-ol, or brassicasterol) from the *E. huxleyi* and the 24-methyl-cholesta-5,24(28)-dien-3β-ol sterol from the *T. pseudonana* culture extracts. Each column consisted of a thin layer of pre-combusted sand (850 °C) on top of 500 mg of Supelclean LC-NH2 (Sigma Aldrich Cat. #57205), wet-packed and conditioned with 6 mL and 8 mL, respectively, of 3:1 (v/v) dichloromethane:isopropyl alcohol (DCM:IPA). The lipid extract, dissolved in 1 mL of DCM:IPA, was emplaced on the SPE column and the NP fraction was eluted with an additional 7 mL of the solvent. The FA fraction was then eluted from the SPE column using 6 mL of 4% acetic acid (v/v) in diethyl ether. A final rinse of the column using 6 mL of methanol was also collected and saved. The lipid fractions were concentrated using a pre-purified stream of N_2_.

The sterol 24-methyl-cholesta-5,24(28)-dien-3β-ol was isolated from the *T. pseudonana* extracts using the non-polar (NP) fraction described above and further separating it using an SPE column packed with 5% deactivated silica gel (0.5 g). The column was eluted sequentially with hexane (4 mL, F1), 1:1 hexane:dichloromethane (4 mL, F2), 4:1 hexane:ethyl ether (4 mL, F3), and methanol (6 mL, F4). The F3 fraction provided sufficiently purified sterol for further isotopic analysis.
