# Supplementary material for: The Influence of Growth Rate on 2H/1H Fractionation in Continuous Cultures of the Coccolithophorid Emiliania huxleyi and the Diatom Thalassiosira pseudonana
Source: PLoS One. 2015 Nov 17;10(11):e0141643. doi: 10.1371/journal.pone.0141643 (PMC4648508; doi:10.1371/journal.pone.0141643)
Supplement: S3 Appendix — (DOCX) [file pone.0141643.s003.docx]

**S.3 Appendix. HPLC-MS purification of alkenones and brassicasterol**

Long-chain di- and tri-unsaturated C_37_ and C_38_ alkenones and brassicasterol were isolated from the NP fraction of the *T. pseudonana* extract using semi-preparative high-performance liquid chromatography - mass spectrometry (HPLC-MS). The technique used was based on the alkenone purification method previously described in [41] modified to facilitate simultaneous isolation of brassicasterol and for optimal utilization of NP lipid SPE fractions from algal culture extracts.

The Agilent HPLC-MS system consisted of an 1100 series HPLC, equipped with a G1379A Degasser, G1311A Quaternary Pump, G1313A ALS Autosampler, G1316A Thermostatted Column Compartment, G1315B Diode Array Detector, and a G1364 Analytical Scale Fraction Collector. The HPLC was equipped with an Alltech (Grace Davison) Prevail Cyano 5 µm, 4.6 x 250 mm analytical column (Grace Davison P/N 99253) and protected by a Prevail Cyano 5 µm, 4.6 mm x 7.5 mm All-Guard cartridge (Grace Davison P/N 99289). Elution of target compounds was monitored using an Agilent G1956B LC/MSD equipped with a G1978A Multimode Ion Source operated in positive atmospheric chemical ionization (APCI+) mode. Agilent LC/MS Chemstation software (Rev. B.02.01-SR2 [Build 260]) was used to control the system and analyze the elution data. All solvents used for HPLC and sample processing were either high-purity OmniSolv HR-GC (EMD Brand) or a similar HPLC-grade.

The HPLC column was held isothermal at 30 °C and preconditioned with 5% DCM in hexane (v/v) at 2.0 mL/min for 1 hour. The NP fractions were dissolved in 100 µL 10% DCM in hexane (v/v) and 50 or 100 µL were injected by the autosampler. The column flow was initiated at 1.0 mL/min of 5% DCM in hexane (v/v). Optimal separation of the individual alkenones and alkenoates was achieved within 40 minutes using these conditions. Brassicasterol was subsequently isolated within an additional 17 minutes using 1.5 mL/min of 40 % DCM (v/v) in hexane. Additional details of the solvent programming are provided in Table 1.

Table 1. Timetable for HPLC solvent gradient elution.

| **Time**  **(min)** | **Solvent A (% v/v)** | **Solvent B**  **(% v/v)** | **Flow Rate**  **(mL/min)** | **Target Compounds/**  **Elution Function** |
| --- | --- | --- | --- | --- |
| 0 | 95 | 5 | 1.0 | **Alkenones, Alkenoates** |
| 40 | 95 | 5 | 1.0 | Solvent Gradient |
| 45 | 60 | 40 | 1.5 | **Brassicasterol** |
| 62 | 60 | 40 | 1.5 | Solvent Gradient |
| 63 | 30 | 70 | 2.0 | Column Cleaning |
| 68 | 30 | 70 | 2.0 | Solvent Gradient |
| 69 | 95 | 5 | 2.0 | Column Reconditioning |
| 74 | 95 | 5 | 2.0 | Flow Rate Decrease |
| 75 | 95 | 5 | 1.0 | Column Ready |

Solvent A: Hexane; Solvent B: Dichloromethane.

A post-column adjustable flow splitter (Analytical Scientific Instruments) with a 100:1 to 5:1 split range directed 3.5 % of the column flow to the MSD for compound detection. A Waters 510 HPLC pump provided a makeup flow of 0.3 mL/min of 2,2,4-trimethylpentane into this stream, improving the ionization and sensitivity of the APCI+ method. The multimode source conditions were: gas temperature, 350 ° C; vaporizer, 250 °C; drying gas, 12.0 L/min; nebulizer pressure, 35 psig; capillary voltage, 2.5 kV; and corona discharge current, 6 µA. Full scan spectra from 200 to 800 daltons were obtained using a 0.10 dalton step-size, 150 threshold, and 140 V fragmentor voltage.

The remainder of the column flow (96.5%) was directed to the fraction collector. Purification of the alkenones from alkenoates was achieved by collecting 30 second intervals of the column elutant in the regions of their expected elution and simultaneously monitoring the APCI-produced protonated M+1 ions (addition of H^+^) of the individual compounds: alkenoates (*m/z* 561, 559, 547), 38:2 alkenone (*m/z* 545), 38:3 alkenone (*m/z* 543), 37:2 alkenone (*m/z* 531), and 37:3 alkenone (*m/z* 529). Collection vials containing the individual alkenones were appropriately combined and quantified, and their purity evaluated by GC-FID. Vials before and after were similarly analyzed to test for full separation and recovery. The elution times observed were: alkenoates (12 – 14 min), 38:2 (16.5 – 18.5 min), 38:3 (19.5 – 21.0 min), 37:2 (29.5 – 32.0 min), and 37:3 (34.5 – 36.0 min). Brassicasterol was similarly isolated using 1 min interval collections while monitoring the M-17 (*m/z* 381) ion (loss of H_2_O and addition of H^+^) during the 56-58 minute elution time.
