# Supplementary material for: The Influence of Growth Rate on 2H/1H Fractionation in Continuous Cultures of the Coccolithophorid Emiliania huxleyi and the Diatom Thalassiosira pseudonana
Source: PLoS One. 2015 Nov 17;10(11):e0141643. doi: 10.1371/journal.pone.0141643 (PMC4648508; doi:10.1371/journal.pone.0141643)
Supplement: S4 Appendix — (DOCX) [file pone.0141643.s004.docx]

**S4 Appendix. Fatty acid and sterol derivatization**

The fatty acids were methylated for both quantification and hydrogen isotope analysis using methanolic HCl, produced by reaction of anhydrous methanol with acetyl chloride (10:1 v/v) in an ice bath. Each polar FA SPE fraction was dissolved in 1 mL of Na_2_SO_4_-dried hexane, and 2 mL of methylating reagent was added. After purging of headspace with pre-purified N_2_, vials were capped securely and heated in a 60 °C oven for 18 hours. After cooling, the reagent was neutralized by the addition of 2 mL of HPLC-grade water and the methylated acids were extracted from the aqueous/methanol layer by 3 additions of 2 mL hexane each. The extracts were combined and back-extracted with 3 mL of HPLC-grade water. The hexane layer containing the methylated acids was carefully removed and dried over pre-combusted Na_2_SO_4_ before subsequent analysis.

The contribution of introduced methyl group hydrogens to the δ^2^H values of the fatty acids was determined by simultaneous methylation of a Na-phthalate standard of known δ^2^H value (-95.5 ± 2.2, Dr. Arndt Schimmelmann, Indiana University). The fatty acid δ^2^H values reported herein have been corrected for the added methyl hydrogens of δ^2^H = -156.2, and represent those of the free carboxylate anions.

Aliquots of each NP SPE fraction containing the alkenones and brassicasterol and each SPE subfraction (silica-gel F3) containing the 24-methyl-cholesta-5,24(28)-dien-3β-ol were silylated to allow for quantification of the sterols and alkenones by GC-FID. After the subsamples were dried under a stream of pre-purified N_2_, 20 µL of fresh pyridine, 20 µL of bis(trimethylsilyl)trifluoracetamide (BSTFA) + 1% trimethylchlorosilane (TMCS), and 20 µL of 5α-cholestane standard in toluene (Na_2_SO_4_-dried) was added as an internal standard to each subsample and the sealed vials were heated for 1 hour at 60 °C. Quantification by GC-FID proceeded immediately thereafter.

Derivatization of brassicasterol and 24-methyl-cholesta-5,24(28)-dien-3β-ol for hydrogen isotope analysis requires a product highly resistant to decomposition and the ability to correct the δ^2^H of the sterol for hydrogens added, neither of which is adequately provided by silylation with BSTFA. The HPLC isolates of brassicasterol and the SPE chromatographic fraction containing the 24-methyl-cholesta-5,24(28)-dien-3β-ol were acetylated with 20 µL of pyridine and 20 µL of acetic anhydride heated in a sealed vial at 70 °C for 30 minutes. The acetic anhydride has a known δ^2^H of -133.2 ± 2.1 (Dr. Arndt Schimmelmann, Indiana University), and the hydrogen isotope values reported for the sterols have been corrected for the hydrogens added by the acetyl group and represent the sterols without the hydroxyl hydrogen atom.
