# Supplementary material for: The Influence of Growth Rate on 2H/1H Fractionation in Continuous Cultures of the Coccolithophorid Emiliania huxleyi and the Diatom Thalassiosira pseudonana
Source: PLoS One. 2015 Nov 17;10(11):e0141643. doi: 10.1371/journal.pone.0141643 (PMC4648508; doi:10.1371/journal.pone.0141643)
Supplement: S5 Appendix — (DOCX) [file pone.0141643.s005.docx]

**S5 Appendix. GC-FID and GC-MS analyses**

The alkenone, sterol, and fatty acid compositions of the continuous cultures were evaluated and quantified by gas chromatography – flame ionization detection (GC-FID). The alkenone and sterol concentrations were obtained by GC-FID analysis of the silylated aliquots (see S4 Appendix) of the respective SPE fractions by comparing their area responses with that of the added 5α-cholestane internal standard. Quantification of fatty acids (as methyl esters) proceeded similarly with aliquots of the methylated products of the FA SPE fractions. The fatty acid concentrations were additionally corrected for procedural recovery (~70%) using a known mass of heneicosanoic acid added to the culture extract before SPE.

Analysis of the HPLC-isolated alkenones and test fractions by GC-FID, with 5α-cholestane internal standard added, provided confirmation of adequate HPLC purification and appropriate dilution amounts for subsequent hydrogen isotope analyses by GC-IRMS. Silylation of test fractions bracketing the HPLC elution time of brassicasterol confirmed that the combined vials contained all of the compound. Aliquots of the acetylated sterols (brassicasterol and 24-methyl-cholesta-5,24(28)-dien-3β-ol ) and methylated fatty acids were similarly quantified to determine the mass isolated and dilution required for GC-IRMS.

GC-FID analysis was conducted on an Agilent 6890N gas chromatograph equipped with an FID, a 7683B Series Automatic Liquid Sampler, a J&W DB5-MS 60 m x 0.32 mm ID (0.25 µm film) column, and helium as carrier gas. Samples were introduced to the column using the PTV inlet in pulsed splitless mode and temperature programmed using: 110 °C (0.85 min hold), ramped at 720 °C/min to 320 °C (2.35 min hold). A final temperature ramp of 720 °C/min to 350°C (5.00 min hold) was used for post-injection cleaning of the inlet. The column oven was programmed using: initial 60 °C, ramped at 15 °C/min to 150 °C, ramped at 6 °C/min to 320 °C (isothermal for 28.00 min). Constant column flow mode at 2.4 mL/min and an initial pressure of 19.22 psig resulted in an average linear velocity of 32 cm/sec. The instrument was controlled and the data processed with GC Chemstation software (Rev. B.01.03 [Build 204]).

Gas chromatography–mass spectrometry (GC-MS) analysis was used for compound identifications, qualitative analysis of the HPLC fractions, and confirmation of GC-FID results. The system used was an Agilent 6890N gas chromatograph coupled with an Agilent 5975 Mass Selective Detector (MSD), and a 7683B Series Automatic Liquid Sampler. The instrument was equipped with split/splitless inlet and a J&W DB5-MS 60 m x 0.32 mm ID (0.25 µm film) column, a 5.0 m guard column, and helium carrier. Samples were analyzed in splitless mode with the injector at 300 °C. Injector purge flow and time were 50 mL/min and 2.00 min, respectively. The column oven was programmed using: initial 60 °C, ramp at 15 °C/min to 150 °C, ramp at 6 °C/min to 320 °C (isothermal for 28.00 min). Constant flow mode at 1.5 mL/min and an initial pressure of 10.73 psig resulted in an average linear velocity of 31 cm/sec. The ion source was operated in electron impact mode with the following: emission current, 35 µA; electron energy, 70 eV; source temperature, 230 °C; quadrupole temperature, 150 °C; transfer line temperature 300 °C; and solvent delay, 8.00 min. The MSD was scanned from 50 to 700 daltons using a sampling of 2^2^ and threshold of 100.
