# Supplementary material for: The Influence of Growth Rate on 2H/1H Fractionation in Continuous Cultures of the Coccolithophorid Emiliania huxleyi and the Diatom Thalassiosira pseudonana
Source: PLoS One. 2015 Nov 17;10(11):e0141643. doi: 10.1371/journal.pone.0141643 (PMC4648508; doi:10.1371/journal.pone.0141643)
Supplement: S6 Appendix — (DOCX) [file pone.0141643.s006.docx]

**S6 Appendix. GC-IRMS Instrumentation for hydrogen isotope analysis of lipids**

The stable hydrogen isotopic compositions of the purified alkenones, brassicasterol, 24-methyl-cholesta-5,24(28)-dien-3β-ol and fatty acids were determined using gas chromatography–isotope ratio mass spectrometry (GC-irMS). The GC-irMS system consisted of a Thermo Electron Corporation Trace GC II gas chromatograph coupled with a Thermo/Finnigan GC-TC High Temperature Conversion Interface (Al_2_O_3_-based pyrolysis unit), which feeds into a Model Delta V Plus irMS, equipped with a Universal 3 +HD collector interface. A TriPlus Autosampler was used for sample introduction into the 325 °C split/splitless inlet of the GC. The Al_2_O_3_-based open pyrolysis tube for the thermal conversion of the GC eluants was held at 1420 °C and preconditioned with multiple injections of hexane, and the H_2_ gas flow to the irMS was through an open split. The GC was outfitted with a J&W DB5-MS 59 m x 0.32 mm ID (0.25 µm film) column, and helium at a constant flow of 1 mL/min was used as carrier gas. The purified compounds/fractions were injected in splitless mode and the GC column was programmed using: initial 80 °C, ramped at 20 °C/min to 200 °C, ramped at 4 °C/min to 320 °C (isothermal for 30.00 min).
